# Promoter methylation of tumor-related genes as a potential biomarker using blood samples for gastric cancer detection

**DOI:** 10.18632/oncotarget.20782

**Published:** 2017-09-08

**Authors:** Jinfeng Wen, Tuo Zheng, Kefeng Hu, Chunxia Zhu, Lihua Guo, Guoliang Ye

**Affiliations:** ^1^ Department of Gastroenterology, The Affiliated Hospital, School of Medicine, Ningbo University, Ningbo, Zhejiang 315020, People's Republic of China; ^2^ Department of Gastroenterology, Ningbo No.1 Hospital, Ningbo, Zhejiang 315000, People's Republic of China

**Keywords:** tumor-related gene, promoter methylation, blood, GC, diagnosis

## Abstract

Gene promoter methylation has been reported in gastric cancer (GC). However, the potential applications of blood-based gene promoter methylation as a noninvasive biomarker for GC detection remain to be evaluated. Hence, we performed this analysis to determine whether promoter methylation of 11 tumor-related genes could become a promising biomarker in blood samples in GC. We found that the cyclin-dependent kinase inhibitor 2A (*p16*), E-cadherin (*CDH1*), runt-related transcription factor 3 (*RUNX3*), human mutL homolog 1 (*MLH1*), RAS association domain family protein 1A (*RASSF1A*), cyclin-dependent kinase inhibitor 2B (*p15*), adenomatous polyposis coli (*APC*), Glutathione S-transferase P1 (*GSTP1*), TP53 dependent G2 arrest mediator candidate (*Reprimo*), and O6-methylguanine-DNAmethyl-transferase (*MGMT*) promoter methylation was notably higher in blood samples of patients with GC compared with non-tumor controls. While death-associated protein kinase (*DAPK*) promoter methylation was not correlated with GC. Further analyses demonstrated that *RUNX3*, *RASSF1A* and *Reprimo* promoter methylation had a good diagnostic capacity in blood samples of GC versus non-tumor controls (*RUNX3*: sensitivity = 63.2% and specificity = 97.5%, *RASSF1A*: sensitivity = 61.5% and specificity = 96.3%, *Reprimo*: sensitivity = 82.0% and specificity = 89.0%). Our findings indicate that promoter methylation of the *RUNX3*, *RASSF1A* and *Reprimo* genes could be powerful and potential noninvasive biomarkers for the detection and diagnosis of GC in blood samples in clinical practices, especially *Reprimo* gene. Further well-designed (multi-center) and prospective clinical studies with large populations are needed to confirm these findings in the future.

## INTRODUCTION

Gastric cancer (GC) is the fifth most common malignant tumor and the third leading cause of death in all human cancers worldwide [1]. Based on GLOBOCAN estimates, approximately 951,600 new cases were clinically diagnosed with GC, leading to about 723,100 deaths in 2012 worldwide [1]. Approximately 70% of GC cases occur in developing countries [2]. Although early detection and treatment have improved survival in early gastric cancer patients, numerous patients with GC are usually diagnosed at advanced stage, with a high mortality rate [3, 4]. Therefore, novel noninvasive and low-cost biomarkers are of importance for early diagnosis and screening of GC.

As a common epigenetic alteration, DNA methylation is associated with human cancers [5–7]. Aberrant DNA methylation of tumor-related genes could be a noninvasive biomarker using body fluid samples (blood or urine etc.) for detecting cancer [8–11]. The cell-cycle inhibitory proteins, tumor suppressor genes (TSGs) *p16*: cyclin-dependent kinase inhibitor 2A (*CDKN2A*) and *p15*: cyclin-dependent kinase inhibitor 2B (*CDKN2B*) are linked to the p53 and retinoblastoma (Rb) pathways [12]. TSG *CDH1* is termed as epithelial cadherin (E-cadherin) or cadherin-1 and is associated with the invasion and metastasis of cancer [13, 14]. Runt-related transcription factor 3 (*RUNX3*) was reported as a new gastric TSG in 2002 and the loss of *RUNX3* expression is related to gastric carcinogenesis [15]. Human mutL homolog 1 (*MLH1*) encoding a DNA mismatch repair (MMR) protein and lack of *MLH1* expression is associated with genomic instability in gastric cancer [16, 17]. RAS association domain family protein 1A (*RASSF1A*) as a TSG has some biological roles in the regulation of cell cycle, microtubule stability, and apoptosis [18]. The adenomatous polyposis coli (*APC*) gene is a TSG involved in multiple functions, including WNT signaling, cell cycle regulation, cell differentiation and proliferation, and transcriptional activation etc. [19]. Glutathione S-transferase P1 (*GSTP1*) is identified as a TSG and has an important function in preventing normal cells against damage by various carcinogens or electrophilic compounds [20, 21]. TP53 dependent G2 arrest mediator candidate (*Reprimo*) is involved in cell cycle regulation [22]. Death-associated protein kinase (*DAPK*), a calcium/calmodulin-dependent serine/threonine kinase, is related to these functions of apoptosis, autophagy, and inflammation [23]. O6-methylguanine-DNAmethyl-transferase (*MGMT*), a DNA repair gene, protects cells against the effects of treatment via eliminating alkyl adducts from the O6-position of guanine [24, 25]. Multiple tumor-related genes are found to be commonly methylated in tissue samples in GC, such as *p16*, *CDH1*, *RUNX3*, *MLH1*, *RASSF1A*, *p15*, *APC*, *DAPK*, *GSTP1*, *Reprimo*, and *MGMT* etc. [22, 26–28].

However, the potential value of the diagnosis of gene promoter methylation in the blood as a promising noninvasive biomarker in GC patients remains to be determined. The purpose of this study was to perform a systematic analysis of all possible candidate genes rather than an individual gene associated with blood-based gene methylation in diagnosing GC.

## RESULTS

### Study characteristics

**Figure 1:**
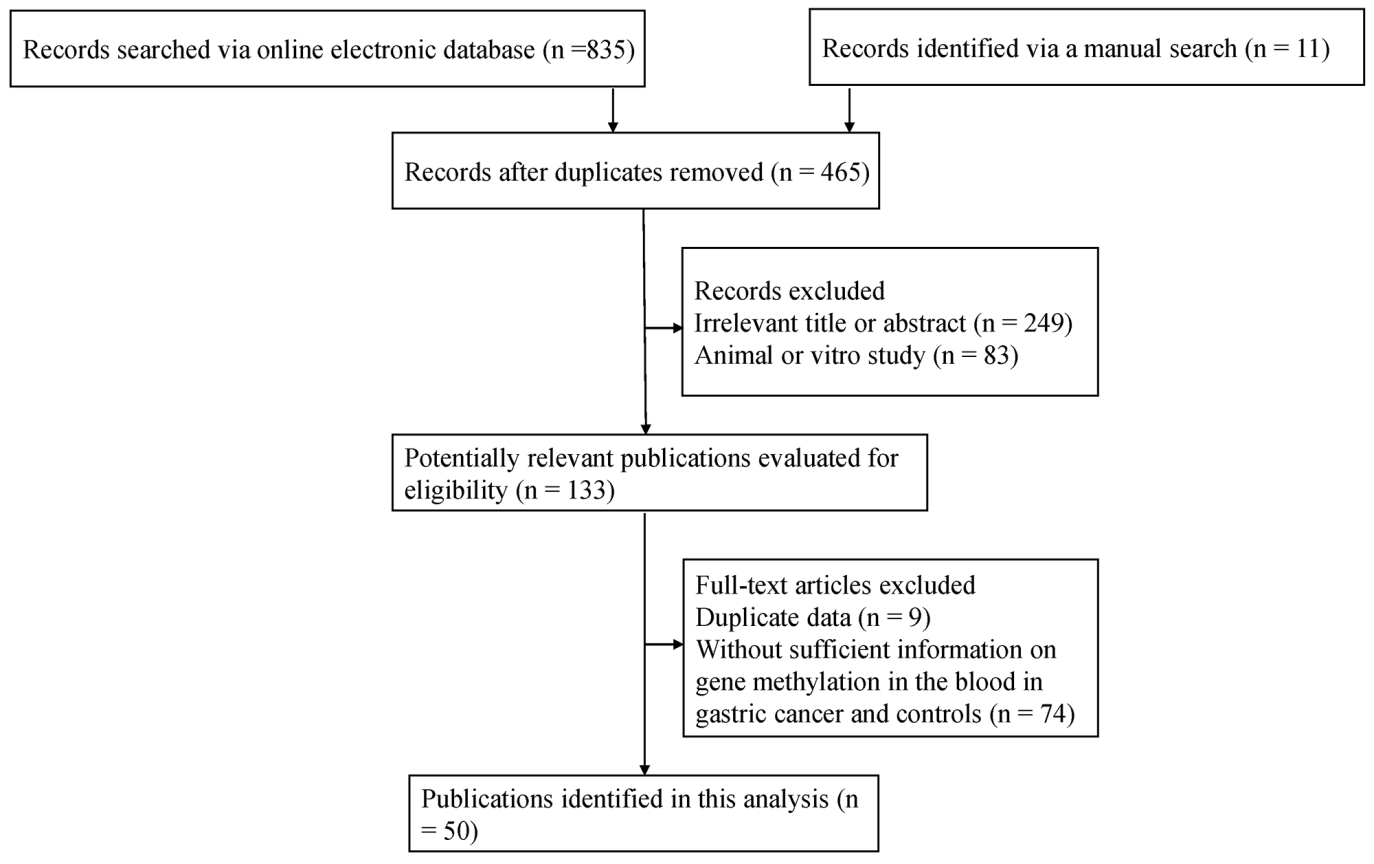
Flow diagram of the search method of the eligible studies in this systematic analysis

According to the described search method, as shown in Figure 1, there were 50 publications published from 2002 to 2017 in the present study. Data involving 51 methylated genes were determined in blood samples of patients with GC from China, Japan, Korea, Iran, Thailand, Chile, Greece, Russia, and Singapore. Supplementary Table 1 lists the detailed characteristics of the eligible studies on blood samples of GC and non-tumor controls. This systematic analysis mainly investigated these methylated genes with more than two studies.

### Gene promoter methylation in the blood in GC

**Figure 2:**
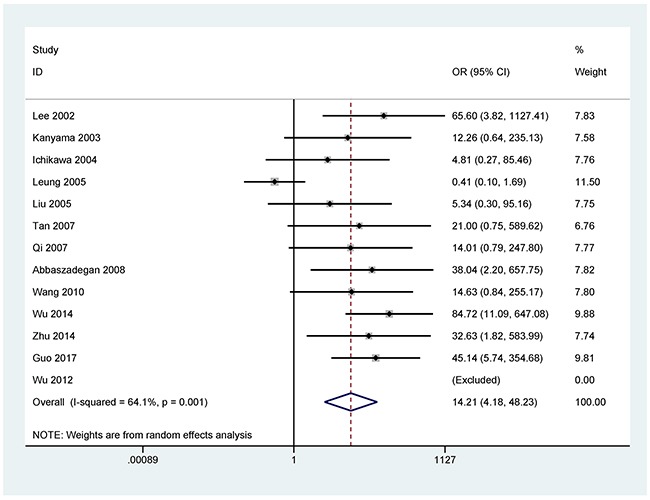
Forest plot of the association between *p16* promotermethylation and GC in blood samples (877 GC patients and 307 non-tumor controls), OR = 14.21, 95% CI = 4.18-48.23, *P* < 0.001, methylation frequency (cancer vs control group): 31.0% vs 2.0%

**Figure 3:**
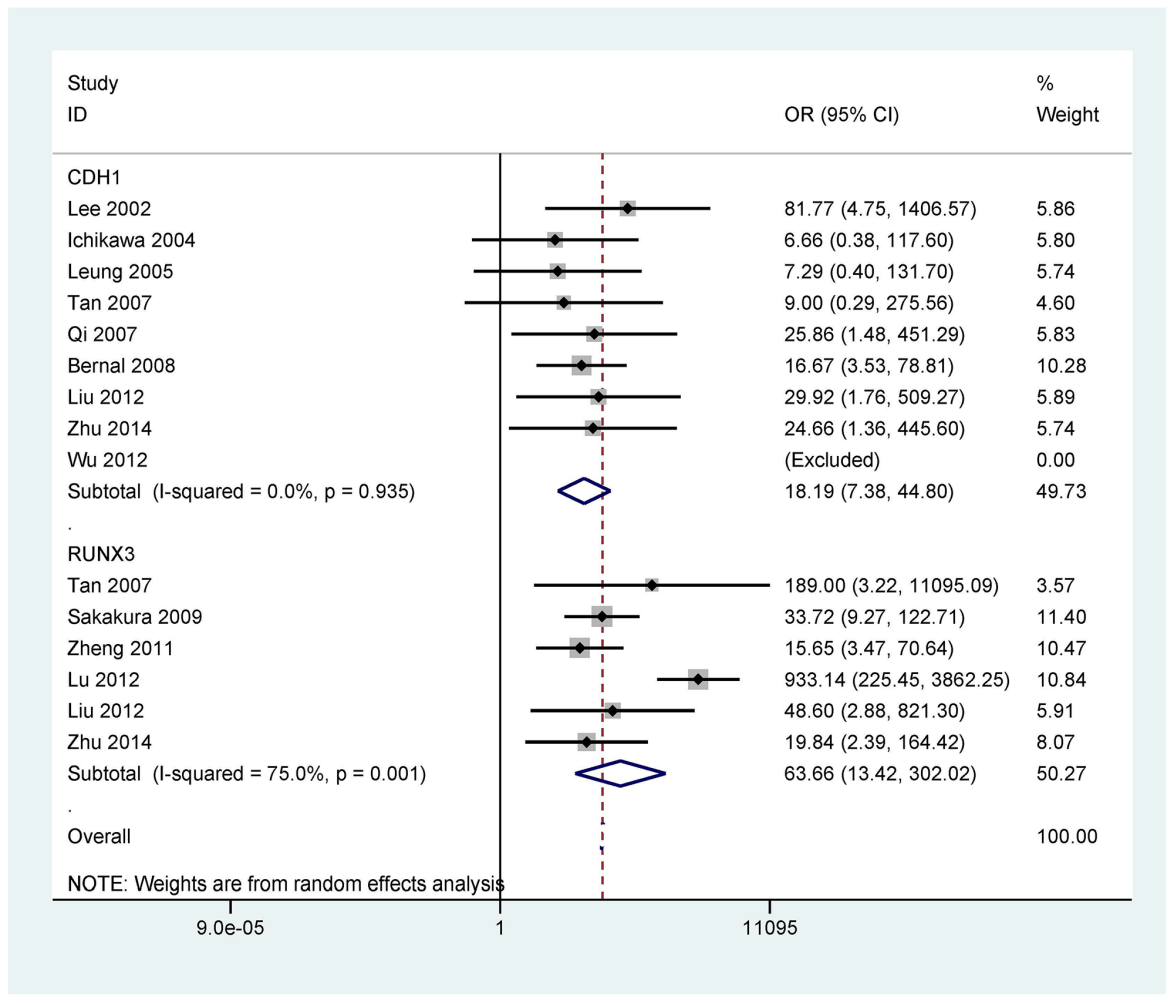
Forest plot of the association between *CDH1* (569 GC patients and 223 non-tumor controls) and *RUNX3* (440 GC patients and 942 non-tumor controls) promoter methylation and GC in blood samples, *CDH1*:OR = 18.19, 95% CI = 7.38-44.80, *P* < 0.001, methylation frequency (cancer vs control group): 24.3% vs 0.9%; *RUNX3*: OR = 63.66, 95% CI = 13.42-302.02, *P* <<0.001, methylation frequency (cancer vs control group): 63.2% vs 2.5%

**Figure 4:**
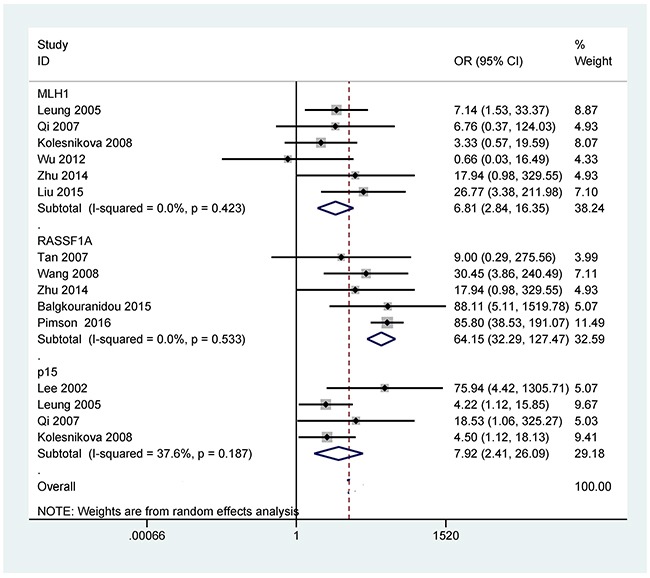
Forest plot of the association between *MLH1* (354 GC patients and 154 non-tumor controls), *RASSF1A* (257 GC patients and 322 non-tumor controls), and *p15* (186 GC patients and 94 non-tumor controls) promoter methylation and GC in blood samples, *MLH1*: OR = 6.81, 95% CI = 2.84-16.35, *P* < 0.001, methylation frequency (cancer vs control group): 19.5% vs 3.2%; *RASSF1A*: OR = 64.15, 95% CI = 32.29-127.47, *P* < 0.001, methylation frequency (cancer vs control group): 61.5% vs 3.7%; and *p15*: OR = 7.92, 95% CI = 2.41-26.09, *P* = 0.001, methylation frequency (cancer vs control group): 43.0% vs 7.4%

**Figure 5:**
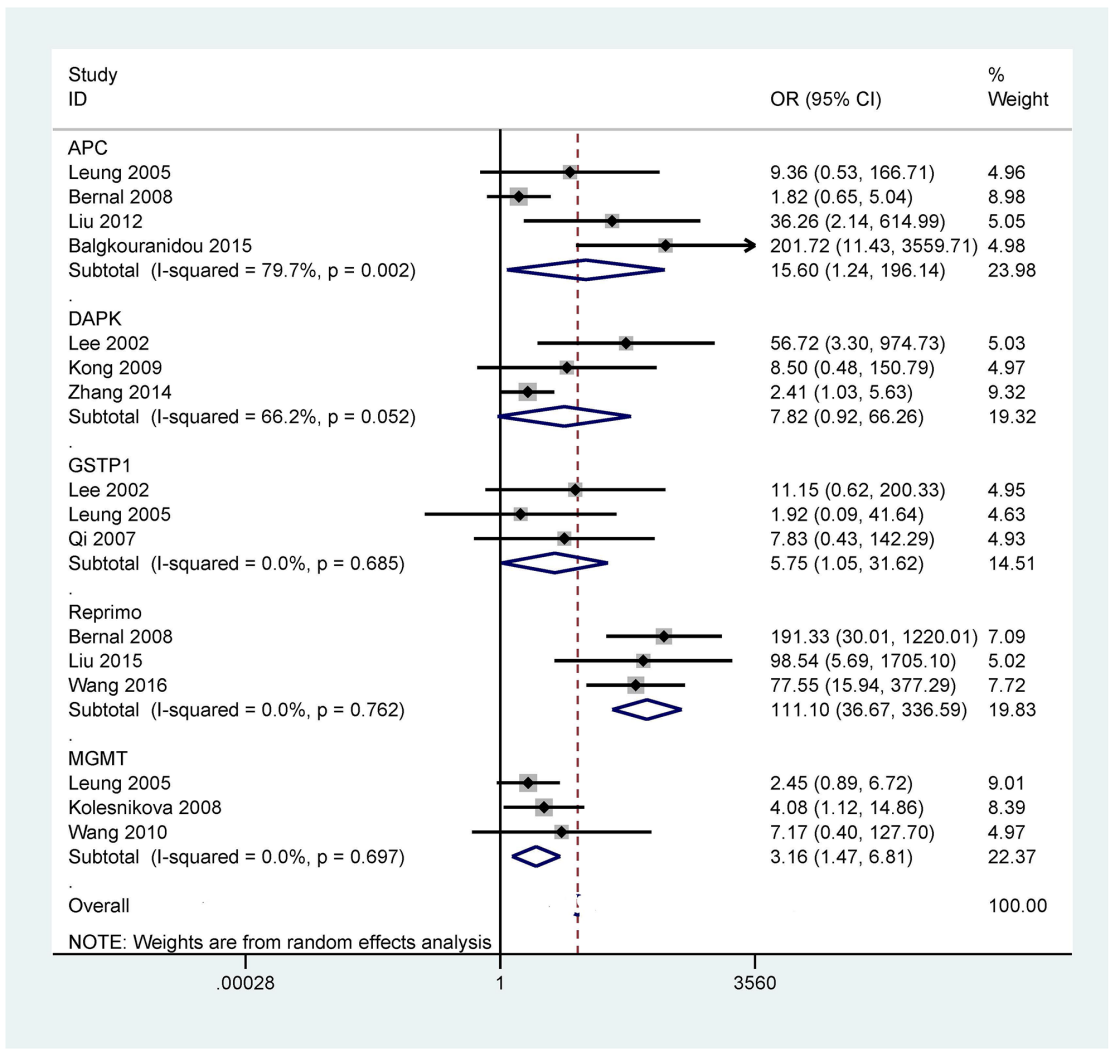
Forest plot of the correlation between *APC* (OR = 15.60, 95% CI = 1.24-196.14, *P* = 0.033, methylation frequency (cancer vs control group): 50.6% vs 17.7%), *DAPK* (OR = 7.82, 95% CI = 0.92-66.26, *P* = 0.059), *GSTP1* (OR = 5.75, 95% CI = 1.05-31.62, *P* = 0.044, methylation frequency (cancer vs control group): 10.8% vs 0.0%), *Reprimo* (OR = 111.10, 95% CI = 36.67-336.59, *P* < 0.001, methylation frequency (cancer vs control group): 82.0% vs 11.0%), and *MGMT* (OR = 3.16, 95% CI = 1.47-6.81, *P* = 0.003, methylation frequency (cancer vs control group): 40.9% vs 26.7%) promoter methylation and GC in the blood

The analyses of more than two studies included 11 tumor-related genes within promoter methylation in blood samples of GC. When GC was compared to non-tumor controls, the results showed that promoter methylation ofthe *p16* (OR = 14.21, 95% CI = 4.18-48.23, *P* < 0.001), *CDH1* (OR = 18.19, 95% CI = 7.38-44.80, *P* < 0.001), *RUNX3* (OR = 63.66, 95% CI = 13.42-302.02, *P* < 0.001), *MLH1* (OR = 6.81, 95% CI = 2.84-16.35, *P* < 0.001), *RASSF1A* (OR = 64.15, 95% CI = 32.29-127.47, *P* < 0.001), *p15* (OR = 7.92, 95% CI = 2.41-26.09, *P* = 0.001), *APC* (OR = 15.60, 95% CI = 1.24-196.14, *P* = 0.033), *GSTP1* (OR = 5.75, 95% CI = 1.05-31.62, *P* = 0.044), *Reprimo* (OR = 111.10, 95% CI = 36.67-336.59, *P* < 0.001), and *MGMT* (OR = 3.16, 95% CI = 1.47-6.81, *P* = 0.003) was significantly correlated with GC in blood samples (Figures 2-5). No significant correlation was found between *DAPK* promoter methylation and GC (OR = 7.82, 95% CI = 0.92-66.26) (Figure 5).

Supplementary Table 2 presents the remaining 40 genes investigated among less than three studies. Of these genes, 20 methylated genes were shown to be significantly associated with GC in the blood (Supplementary Table 2), additional studies with large populations should be done to confirm the results of gene methylation with fewer than three studies.

### Subgroup analysis of *p16* promoter methylation

According to the eligible subgroups, a subgroup analysis of testing method ((methylation specific PCR (MSP) and non-methylation specific PCR (Non-MSP)) was conducted in *p16* promoter methylation (Supplementary Figure 1). The result demonstrated that *p16* promoter methylation was correlated with GC in the MSP method (OR = 20.88, 95% CI = 8.28-52.64, *P* < 0.001), but not in the Non-MSP method (OR = 7.46, 95% CI = 0.15-360.60, *P* = 0.310).

### Sensitivity analyses

For the results with substantial heterogeneity among more than two studies (*p16*, *RUNX3*, and *APC* genes: *P* < 0.1), we conducted sensitivity analyses to determine the stability of the overall OR and the change of heterogeneity based on the omission of a single study.

When one study (Leung 2005 *et al*) [29] was removed in *p16* promoter methylation, the pooled OR of *p16* promoter methylation was 25.31 (95% CI = 11.29-56.76, *P* < 0.001), while heterogeneity was significantly reduced (*P* = 0.880).

The OR value of *RUNX3* promoter methylation was 27.28 (95% CI = 11.89-62.62, *P* < 0.001) by removing a single study by Lu 2012 *et al* [30], which caused the absence of heterogeneity (*P* = 0.783).

When we removed this study by Bernal 2008 *et al* [22], and re-calculated the combined OR value of *APC* promoter methylation (OR = 40.98, 95% CI = 7.25-231.79, *P* < 0.001), resulting in a dramatically decreased heterogeneity (*P* = 0.333).

### Publication bias

**Figure 6:**
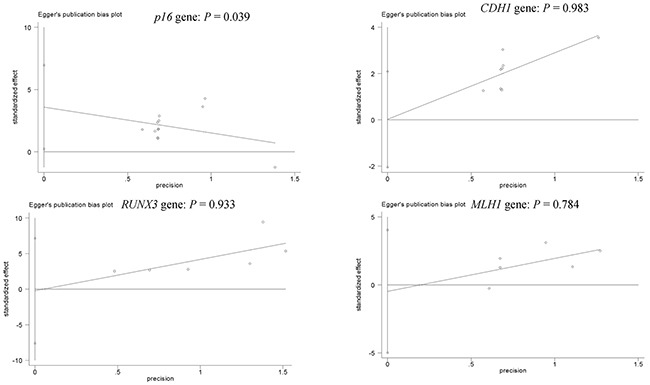
Forest plot of publication bias using Egger's test in the *p16* (cancer vs control group: *P* = 0.039 < 0.05), *CDH1*, *RUNX3*, and *MLH1*genes (cancer vs control group: all *Ps* > 0.05)

Egger's test was used to measure the potential publication bias for the results with greater than five studies (*p16*, *CDH1*, *RUNX3*, and *MLH1* genes). Figure 6 shows the statistical data of funnel plot symmetry, which indicted the absence of publication bias regarding promoter methylation of the *CDH1*, *RUNX3*, and *MLH1*genes (*P* > 0.05). There was a slight publication bias for *p16* promoter methylation (*P* = 0.039 < 0.05).

### Gene promoter methylation in relation to clinicopathological features of GC

We analyzed other clinical effects of *p16*, *RASSF1A*, *DAPK*, and *p15* promoter methylation with the clinicopathological characteristics of patients with GC in the blood. *p16* promoter methylation was not correlated with gender, age and lymph node status (*P* > 0.1) (Supplementary Figure 2), while a positive relationship was found between *p16* promoter methylation and clinical stage (OR = 2.21, 95% CI = 1.10-4.42, *P* = 0.025) (Supplementary Figure 2).

No correlation was found between *RASSF1A*, *DAPK*, and *p15* promoter methylation and clinicopathological features of GC (Supplementary Figure 3) (all *Ps* > 0.1), including gender and age for *RASSF1A* promoter methylation; gender and lymph node status for *p15* promoter methylation; and gender, clinical stage or lymph node status for *DAPK* promoter methylation. Because the sample sizes of the correlation of gene promoter methylation with clinicopathological characteristics were small in blood samples of GC patients. These results should be cautious.

## DISCUSSION

Increasing evidence demonstrated the use of promoter methylation of some tumor-related genes as a powerful noninvasive biomarker for the detection and diagnosis of cancer in the blood in clinical settings [31–33]. However, the diagnostic role of promoter methylation status of the tumor-related genes in blood samples of patients with GC lacks quantitative assessment. Therefore, an integrated analysis was performed to evaluate the ability of 11 tumor-related genes (*p16*, *CDH1*, *RUNX3*, *MLH1*, *RASSF1A*, *p15*, *APC*, *DAPK*, *GSTP1*, *Reprimo*, and *MGMT*) promoter methylation test using blood samples as a feasible biomarker in GC diagnosis and screening.

Some studies reported that promoter methylation of the *p16*, *CDH1*, *RUNX3*, *MLH1*, *RASSF1A*, *p15*, *APC*, *DAPK*, *GSTP1*, *Reprimo*, and *MGMT* was frequent in the blood in patients with GC [34–38]. However, the results of the frequencies of promoter methylation of these 11 tumor-related genes are conflicting and different in blood samples of GC patients and non-tumor controls. For example, Wu 2012 *et al* reported that no promoter methylation of the *p16* gene was detected in GC and non-tumor controls [39]. While Leung 2005 *et al* reported that *p16* promoter methylation in GC was significantly lower than in controls (8.3% vs 18.2%) [29]. Promoter methylation of the *P16* gene had a higher level in GC than in controls among numerous studies [38, 40–43]. *CDH1* promoter methylation showed a frequency from 0% [39] to 57.4% [38] in blood samples of patients with GC, and a frequency from 0% [39] to 6.5% [22] in normal controls. Tan 2007 *et al* reported that *RUNX3* promoter methylation was found in all four GC patients and was not detected in 10 controls [44]. Some studies demonstrated that the frequency of *RUNX3* promoter methylation ranged from 37.3% [45] to 95.4% [36] in blood samples of GC patients. Moreover, only Sakakura 2009 *et al* recorded that *RUNX3* promoter methylation had a frequency of > 5% in non-tumor controls [36].

The frequency of *MLH1* promoter methylation was not very high in blood samples of patients with GC, with a range from 0.7% [39] to 48% [46]. *RASSF1A* gene within the promoter had a low methylation rate (34%) in the blood in GC by Wang 2008 *et al* [47], on the other hand, Pimson 2016 *et al* reported that *RASSF1A* gene within the promoter had a high methylation level (83.2%) in blood samples of GC [48]. *p15* gene within the promoter was methylated with a frequency of < 60% in blood samples of patients with GC [29, 37, 38]. *APC* promoter methylation had a similar and high level in the blood of GC and controls (76.7% vs 64.5%) [22]. While *APC* promoter methylation showed a low level in blood samples of GC and controls (16.7% vs 0%) by Leung 2005 *et al* [29]. *DAPK* gene within the promoter was found to be methylated among both GC and controls (49.1% vs 28.6%) [49]. While only promoter methylation of the *DAPK* gene was reported in GC by Lee 2002 *et al* [38]. *GSTP1* promoter methylation had a frequency of < 20% in blood samples of GC patients [29, 38]. *Reprimo* promoter methylation had a high rate in blood samples of patients with GC (> 60%) [22, 34, 46]. Leung 2005 *et al* showed no correlation between *MGMT* promoter methylation and GC [29]. A significant correlation was reported between *MGMT* promoter methylation and GC by Kolesnikova 2008 *et al* [37].

The current analyses comprising all eligible articles revealed that promoter methylation of the *p16*, *CDH1*, *RUNX3*, *MLH1*, *RASSF1A*, *p15*, *APC*, *GSTP1*, *Reprimo*, and *MGMT* was significantly higher in blood samples of patients with GC compared with non-tumor controls. But *DAPK* promoter methylation had a similar frequency in the blood in GC and controls, these results suggested that promoter methylation of the ten tumor-related genes (*p16*, *CDH1*, *RUNX3*, *MLH1*, *RASSF1A*, *p15*, *APC*, *GSTP1*, *Reprimo*, and *MGMT*) may be potential biomarkers based-blood test for GC. In our study, we found different methylation frequencies of the ten tumor-related genes in blood samples in GC vs controls (*p16*: 31.0% vs 2.0%, *CDH1*: 24.3% vs 0.9%, *RUNX3*: 63.2% vs 2.5%, *MLH1*: 19.5% vs 3.2%, *RASSF1A*: 61.5% vs 3.7%, *p15*: 43.0% vs 7.4%, *APC*: 50.6% vs 17.7%, *GSTP1*:10.8% vs 0.0%, *Reprimo*: 82.0% vs 11.0%, and *MGMT*: 40.9% vs 26.7%) (Supplementary Table 2).

Numerous studies suggested that promoter methylationof the *RUNX3*, *RASSF1A* and *Reprimo* genes was more common in GC tissues compared with normal tissues, suggesting that promoter methylation of the *RUNX3*, *RASSF1A*, and *Reprimo* genes may induce the tumorigenesis of gastric cancer [50–53]. Additionally, *RASSF1A* promoter methylation had a frequency of 93% in blood samples of colorectal cancer and a frequency of 61.6% in non-cancer controls [54], but had a low frequency in lung cancer (37.1%) [55] and nasopharyngeal carcinoma (19%) in the blood [5]. *RUNX3* promoter methylation was shown to have a frequency of 43.9% in blood samples of lung cancer [55]. Nishio *et al* reported that *RUNX3* promoter methylation showed a frequency of 29% in the blood of a large colorectal cancer patients (344 cases) [56]. *Reprimo* methylation was not frequently studied in blood samples of other human cancers. Ellinger *et al* reported that *Reprimo* promoter methylation had a very low frequency (1.2%) in blood samples of prostate cancer [57]. The current analyses indicated that the three *RUNX3*, *RASSF1A* and *Reprimo* genes promoter methylation had better diagnostic capacity (*RUNX3*: sensitivity = 63.2% and specificity = 97.5%, *RASSF1A*: sensitivity = 61.5% and specificity = 96.3%, *Reprimo*: sensitivity = 82.0% and specificity = 89.0%), which suggested that the three tumor-related genes could become potential noninvasive biomarkers using blood samples for the early detection of GC, particularly *Reprimo* promoter methylation.

When GC was compared to non-tumor controls in the blood, significant heterogeneity was detected in the *p16*, *RUNX3*, and *APC* genes (*P* < 0.1). When we removed one study (Leung 2005 *et al*) [29] in *p16* promoter methylation, this study by Lu 2012 *et al* [30] in *RUNX3* promoter methylation, and one study by Bernal 2008 *et al* [22] in *APC* promoter methylation. The re-calculated OR remained significant in the sensitivity analyses, with no obvious evidence of heterogeneity (*P* > 0.1). The reasons for the observed bias were not very clear, perhaps due to the use of inappropriate or different conditions in methylation detection.

Several limitations of the present study should be considered. First, our study mainly included Asian population, and partly consisted of Caucasian population. While Africans were lacking. Second, a slight evidence of publication bias was measured in the *p16* gene, only eligible articles published in English or Chinese were included in our analysis. Other papers in other languages and unpublished studies and conference abstracts, were excluded because of the insufficient information, which may result in a slight bias. Third, the sample sizes and studies of gene promoter methylation with clinicopathological features were small. More well-designed studies with large sample sizes, detained clinical stages and prognostic analysis are needed in blood samples of GC in the future. Finally, gene methylation with fewer than three studies should be further done in large populations.

In conclusion, the current findings reveal that *p16*, *CDH1*, *RUNX3*, *MLH1*, *RASSF1A*, *p15*, *APC*, *GSTP1*, *Reprimo*, or *MGMT*promoter methylation is associated with blood samples of patients with GC. *RUNX3*, *RASSF1A* and *Reprimo* promoter methylation may be potential useful noninvasive biomarkers for the detection of GC, but other *p16*, *CDH1*, *MLH1*, *p15*, *APC*, *GSTP1*, and *MGMT* genes show a low diagnostic effect for GC in blood samples. Additional clinical prospective researches with larger populations of blood samples are essential to further validate the diagnostic and screening value of the *RUNX3*, *RASSF1A* and *Reprimo* promoter methylation in GC.

## MATERIALS AND METHODS

### Search strategy

PubMed, EMBASE, EBSCO, Wanfang, and CNKI databases were searched to find eligible publications prior to April 28, 2017. We used the following key words and free terms: (stomach OR gastric) AND (cancer OR tumor OR neoplasm OR carcinoma) AND (blood OR serum OR sera OR plasma) AND (methylation OR methylated OR hypermethylation OR epigenetic silencing OR epigenetic inactivation). A manual search from the reference lists of the included studies was performed for other potential articles.

### Study selection

The eligible studies were selected if they satisfied the inclusion criteria: 1) the patients were limited to the diagnosis of GC; 2) case-control or cohort studies reported the information between gene promoter methylation and GC in blood samples; 3) studies provided sufficient data to assess the difference of gene promoter methylation between GC and controls without cancer, and the correlation of gene promoter methylation with clinicopathological features of GC. Only the article with the most detailed information was chosen when the overlapping study populations were reported in several papers published.

### Data extraction

The following data were extracted using a standardized form: first author's surname, year of publication, study location, ethnicity, mean or median age, methylation detect methods, cancer stage, sample size of cases and controls, control types, and GC patients’ characteristics, such as gender (male vs female), age (≥ 60 years vs < 60 years), clinical stage (stage 3-4 vs stage 1-2) and lymph node status (positive vs negative).

### Data analysis

The pooled data were analyzed using Stata software (version 12.0, Stata Corporation, College Station, TX, USA). The overall odds ratios (ORs) with the corresponding 95% confidence intervals (95% CIs) were calculated to evaluate the relationship of gene promoter methylation between GC and controls in blood samples. Moreover, the correlation of gene promoter methylation with clinicopathological characteristics of GC was performed among more than one study. The Cochran's Q statistic was used to estimate the possible heterogeneity among the included studies [58]. The random-effects model was chosen to make the results more reliable in the meta-analysis. When there was substantial heterogeneity (*P* < 0.1) for the results with more than two studies, we conducted a sensitivity analysis of the omission of an individual study to assess the change of the recalculated OR and heterogeneity [59, 60]. The possible publication bias was estimated using Egger's test for methylated genes with more than five studies [61].

## SUPPLEMENTARY MATERIALS FIGURES AND TABLES







## References

[1] Torre LA, Bray F, Siegel RL, Ferlay J, Lortet-Tieulent J, Jemal A (2015). Global cancer statistics, 2012. CA Cancer J Clin.

[2] Ferlay J, Soerjomataram I, Dikshit R, Eser S, Mathers C, Rebelo M, Parkin DM, Forman D, Bray F (2015). Cancer incidence and mortality worldwide: sources, methods and major patterns in GLOBOCAN 2012. Int J Cancer.

[3] Oba K, Paoletti X, Bang YJ, Bleiberg H, Burzykowski T, Fuse N, Michiels S, Morita S, Ohashi Y, Pignon JP, Rougier P, Sakamoto J, GASTRIC (Global Advanced/Adjuvant Stomach Tumor Research International Collaboration) Group (2013). Role of chemotherapy for advanced/recurrent gastric cancer: an individual-patient-data meta-analysis. Eur J Cancer.

[4] Hartgrink HH, Jansen EP, van Grieken NC, van de Velde CJ (2009). Gastric cancer. Lancet.

[5] Ye M, Huang T, Ni C, Yang P, Chen S (2017). Diagnostic capacity of RASSF1A promoter methylation as a biomarker in tissue, brushing, and blood samples of nasopharyngeal carcinoma. EBioMedicine.

[6] Pisanic TR, Athamanolap P, Wang TH (2017). Defining, distinguishing and detecting the contribution of heterogeneous methylation to cancer heterogeneity. Semin Cell Dev Biol.

[7] Zhao R, Choi BY, Lee MH, Bode AM, Dong Z (2016). Implications of genetic and epigenetic alterations of CDKN2A (p16(INK4a)) in cancer. EBioMedicine.

[8] Wielscher M, Vierlinger K, Kegler U, Ziesche R, Gsur A, Weinhausel A (2015). Diagnostic performance of plasma DNA methylation profiles in lung cancer, pulmonary fibrosis and COPD. EBioMedicine.

[9] Nagata S, Hamada T, Yamada N, Yokoyama S, Kitamoto S, Kanmura Y, Nomura M, Kamikawa Y, Yonezawa S, Sugihara K (2012). Aberrant DNA methylation of tumor-related genes in oral rinse: a noninvasive method for detection of oral squamous cell carcinoma. Cancer.

[10] Renard I, Joniau S, van Cleynenbreugel B, Collette C, Naome C, Vlassenbroeck I, Nicolas H, de Leval J, Straub J, Van Criekinge W, Hamida W, Hellel M, Thomas A (2010). Identification and validation of the methylated TWIST1 and NID2 genes through real-time methylation-specific polymerase chain reaction assays for the noninvasive detection of primary bladder cancer in urine samples. Eur Urol.

[11] Ni S, Ye M, Huang T (2017). Short stature homeobox 2 methylation as a potential noninvasive biomarker in bronchial aspirates for lung cancer diagnosis. Oncotarget.

[12] Furonaka O, Takeshima Y, Awaya H, Ishida H, Kohno N, Inai K (2004). Aberrant methylation of p14(ARF), p15(INK4b) and p16(INK4a) genes and location of the primary site in pulmonary squamous cell carcinoma. Pathol Int.

[13] Canel M, Serrels A, Frame MC, Brunton VG (2013). E-cadherin-integrin crosstalk in cancer invasion and metastasis. J Cell Sci.

[14] Li L, Hartley R, Reiss B, Sun Y, Pu J, Wu D, Lin F, Hoang T, Yamada S, Jiang J, Zhao M (2012). E-cadherin plays an essential role in collective directional migration of large epithelial sheets. Cell Mol Life Sci.

[15] Li QL, Ito K, Sakakura C, Fukamachi H, Inoue K, Chi XZ, Lee KY, Nomura S, Lee CW, Han SB, Kim HM, Kim WJ, Yamamoto H (2002). Causal relationship between the loss of RUNX3 expression and gastric cancer. Cell.

[16] Ottini L, Falchetti M, Lupi R, Rizzolo P, Agnese V, Colucci G, Bazan V, Russo A (2006). Patterns of genomic instability in gastric cancer: clinical implications and perspectives. Ann Oncol.

[17] Leung SY, Yuen ST, Chung LP, Chu KM, Chan AS, Ho JC (1999). hMLH1 promoter methylation and lack of hMLH1 expression in sporadic gastric carcinomas with high-frequency microsatellite instability. Cancer Res.

[18] Agathanggelou A, Cooper WN, Latif F (2005). Role of the Ras-association domain family 1 tumor suppressor gene in human cancers. Cancer Res.

[19] Shen C, Sheng Q, Zhang X, Fu Y, Zhu K (2016). Hypermethylated APC in serous carcinoma based on a meta-analysis of ovarian cancer. J Ovarian Res.

[20] Masood N, Kayani MA (2013). Expression patterns of carcinogen detoxifying genes (CYP1A1, GSTP1 & GSTT1) in HNC patients. Pathol Oncol Res.

[21] Hayes JD, Flanagan JU, Jowsey IR (2005). Glutathione transferases. Annu Rev Pharmacol Toxicol.

[22] Bernal C, Aguayo F, Villarroel C, Vargas M, Diaz I, Ossandon FJ, Santibanez E, Palma M, Aravena E, Barrientos C, Corvalan AH (2008). Reprimo as a potential biomarker for early detection in gastric cancer. Clin Cancer Res.

[23] Huang Y, Chen L, Guo L, Hupp TR, Lin Y (2014). Evaluating DAPK as a therapeutic target. Apoptosis.

[24] Shibata T, Glynn N, McMurry TB, McElhinney RS, Margison GP, Williams DM (2006). Novel synthesis of O6-alkylguanine containing oligodeoxyribonucleotides as substrates for the human DNA repair protein, O6-methylguanine DNA methyltransferase (MGMT). Nucleic Acids Res.

[25] Esteller M, Garcia-Foncillas J, Andion E, Goodman SN, Hidalgo OF, Vanaclocha V, Baylin SB, Herman JG (2000). Inactivation of the DNA-repair gene MGMT and the clinical response of gliomas to alkylating agents. N Engl J Med.

[26] Kupcinskaite-Noreikiene R, Ugenskiene R, Noreika A, Rudzianskas V, Gedminaite J, Skieceviciene J, Juozaityte E (2016). Gene methylation profile of gastric cancerous tissue according to tumor site in the stomach. BMC Cancer.

[27] Zou XP, Zhang B, Zhang XQ, Chen M, Cao J, Liu WJ (2009). Promoter hypermethylation of multiple genes in early gastric adenocarcinoma and precancerous lesions. Hum Pathol.

[28] Tamura G (2004). Promoter methylation status of tumor suppressor and tumor-related genes in neoplastic and non-neoplastic gastric epithelia. Histol Histopathol.

[29] Leung WK, To KF, Chu ES, Chan MW, Bai AH, Ng EK, Chan FK, Sung JJ (2005). Potential diagnostic and prognostic values of detecting promoter hypermethylation in the serum of patients with gastric cancer. Br J Cancer.

[30] Lu XX, Yu JL, Ying LS, Han J, Wang S, Yu QM, Wang XB, Fang XH, Ling ZQ (2012). Stepwise cumulation of RUNX3 methylation mediated by Helicobacter pylori infection contributes to gastric carcinoma progression. Cancer.

[31] Ye M, Huang T, Ying Y, Li J, Yang P, Ni C, Zhou C, Chen S (2017). Detection of 14-3-3 sigma (σ) promoter methylation as a noninvasive biomarker using blood samples for breast cancer diagnosis. Oncotarget.

[32] Potter NT, Hurban P, White MN, Whitlock KD, Lofton-Day CE, Tetzner R, Koenig T, Quigley NB, Weiss G (2014). Validation of a real-time PCR-based qualitative assay for the detection of methylated SEPT9 DNA in human plasma. Clin Chem.

[33] Hoque MO, Begum S, Topaloglu O, Jeronimo C, Mambo E, Westra WH, Califano JA, Sidransky D (2004). Quantitative detection of promoter hypermethylation of multiple genes in the tumor, urine, and serum DNA of patients with renal cancer. Cancer Res.

[34] Wang H, Zheng Y, Lai J, Luo Q, Ke H, Chen Q (2016). Methylation-sensitive melt curve analysis of the reprimo gene methylation in gastric cancer. PLoS One.

[35] Balgkouranidou I, Matthaios D, Karayiannakis A, Bolanaki H, Michailidis P, Xenidis N, Amarantidis K, Chelis L, Trypsianis G, Chatzaki E, Lianidou ES, Kakolyris S (2015). Prognostic role of APC and RASSF1A promoter methylation status in cell free circulating DNA of operable gastric cancer patients. Mutat Res.

[36] Sakakura C, Hamada T, Miyagawa K, Nishio M, Miyashita A, Nagata H, Ida H, Yazumi S, Otsuji E, Chiba T, Ito K, Ito Y (2009). Quantitative analysis of tumor-derived methylated RUNX3 sequences in the serum of gastric cancer patients. Anticancer Res.

[37] Kolesnikova EV, Tamkovich SN, Bryzgunova OE, Shelestyuk PI, Permyakova VI, Vlassov VV, Tuzikov AS, Laktionov PP, Rykova EY (2008). Circulating DNA in the blood of gastric cancer patients. Ann N Y Acad Sci.

[38] Lee TL, Leung WK, Chan MW, Ng EK, Tong JH, Lo KW, Chung SC, Sung JJ, To KF (2002). Detection of gene promoter hypermethylation in the tumor and serum of patients with gastric carcinoma. Clin Cancer Res.

[39] Wu PY, Zhang Z, Wang JM, Guo WW, Xiao N, He Q, Wang YP, Fan YM (2012). Germline promoter hypermethylation of tumor suppressor genes in gastric cancer. World J Gastroenterol.

[40] Guo L, Huang C, Ji QJ (2017). Aberrant promoter hypermethylation of p16, survivin, and retinoblastoma in gastric cancer. Bratisl Lek Listy.

[41] Wu YC, Lv P, Han J, Yu JL, Zhu X, Hong LL, Zhu WY, Yu QM, Wang XB, Li P, Ling ZQ (2014). Enhanced serum methylated p16 DNAs is associated with the progression of gastric cancer. Int J Clin Exp Pathol.

[42] Abbaszadegan MR, Moaven O, Sima HR, Ghafarzadegan K, A’Rabi A, Forghani MN, Raziee HR, Mashhadinejad A, Jafarzadeh M, Esmaili-Shandiz E, Dadkhah E (2008). p16 promoter hypermethylation: a useful serum marker for early detection of gastric cancer. World J Gastroenterol.

[43] Kanyama Y, Hibi K, Nakayama H, Kodera Y, Ito K, Akiyama S, Nakao A (2003). Detection of p16 promoter hypermethylation in serum of gastric cancer patients. Cancer Sci.

[44] Tan SH, Ida H, Lau QC, Goh BC, Chieng WS, Loh M, Ito Y (2007). Detection of promoter hypermethylation in serum samples of cancer patients by methylation-specific polymerase chain reaction for tumour suppressor genes including RUNX3. Oncol Rep.

[45] Liu JB, Wu XM, Cai J, Zhang JY, Zhang JL, Zhou SH, Shi MX, Qiang FL (2012). CpG island methylator phenotype and Helicobacter pylori infection associated with gastric cancer. World J Gastroenterol.

[46] Liu L, Yang X (2015). Implication of Reprimo and hMLH1 gene methylation in early diagnosis of gastric carcinoma. Int J Clin Exp Pathol.

[47] Wang YC, Yu ZH, Liu C, Xu LZ, Yu W, Lu J, Zhu RM, Li GL, Xia XY, Wei XW, Ji HZ, Lu H, Gao Y (2008). Detection of RASSF1A promoter hypermethylation in serum from gastric and colorectal adenocarcinoma patients. World J Gastroenterol.

[48] Pimson C, Ekalaksananan T, Pientong C, Promthet S, Putthanachote N, Suwanrungruang K, Wiangnon S (2016). Aberrant methylation of PCDH10 and RASSF1A genes in blood samples for non-invasive diagnosis and prognostic assessment of gastric cancer. PeerJ.

[49] Zhang X, Zhang X, Sun B, Lu H, Wang D, Yuan X, Huang Z (2014). Detection of aberrant promoter methylation of RNF180, DAPK1 and SFRP2 in plasma DNA of patients with gastric cancer. Oncol Lett.

[50] Fan XY, Hu XL, Han TM, Wang NN, Zhu YM, Hu W, Ma ZH, Zhang CJ, Xu X, Ye ZY, Han CM, Pan WS (2011). Association between RUNX3 promoter methylation and gastric cancer: a meta-analysis. BMC Gastroenterol.

[51] Liu X, Wang L, Guo Y (2016). The association between runt-related transcription factor 3 gene promoter methylation and gastric cancer: a meta-analysis. J Cancer Res Ther.

[52] Ooki A, Yamashita K, Yamaguchi K, Mondal A, Nishimiya H, Watanabe M (2013). DNA damage-inducible gene, reprimo functions as a tumor suppressor and is suppressed by promoter methylation in gastric cancer. Mol Cancer Res.

[53] Shi DT, Han M, Gao N, Tian W, Chen W (2014). Association of RASSF1A promoter methylation with gastric cancer risk: a meta-analysis. Tumour Biol.

[54] Cassinotti E, Melson J, Liggett T, Melnikov A, Yi Q, Replogle C, Mobarhan S, Boni L, Segato S, Levenson V (2012). DNA methylation patterns in blood of patients with colorectal cancer and adenomatous colorectal polyps. Int J Cancer.

[55] Wang BH, Li YY, Han JZ, Zhou LY, Lv YQ, Zhang HL, Zhao L (2017). Gene methylation as a powerful biomarker for detection and screening of non-small cell lung cancer in blood. Oncotarget.

[56] Nishio M, Sakakura C, Nagata T, Komiyama S, Miyashita A, Hamada T, Kuryu Y, Ikoma H, Kubota T, Kimura A, Nakanishi M, Ichikawa D, Fujiwara H (2010). RUNX3 promoter methylation in colorectal cancer: its relationship with microsatellite instability and its suitability as a novel serum tumor marker. Anticancer Res.

[57] Ellinger J, Haan K, Heukamp LC, Kahl P, Buttner R, Muller SC, von Ruecker A, Bastian PJ (2008). CpG island hypermethylation in cell-free serum DNA identifies patients with localized prostate cancer. Prostate.

[58] Coory MD (2010). Comment on: Heterogeneity in meta-analysis should be expected and appropriately quantified. Int J Epidemiol.

[59] Higgins JP, Thompson SG, Deeks JJ, Altman DG (2003). Measuring inconsistency in meta-analyses. BMJ.

[60] Lau J, Ioannidis JP, Schmid CH (1997). Quantitative synthesis in systematic reviews. Ann Intern Med.

[61] Egger M, Davey Smith G, Schneider M, Minder C (1997). Bias in meta-analysis detected by a simple, graphical test. BMJ.

