# Supplementary material for: Promoter methylation of tumor-related genes as a potential biomarker using blood samples for gastric cancer detection
Source: Oncotarget. 2017 Sep 8;8(44):77783–93. doi: 10.18632/oncotarget.20782 (PMC5652815; doi:10.18632/oncotarget.20782)
Supplement: Supplementary file 1 [file oncotarget-08-77783-s001.pdf]

Promoter methylation of tumor-related genes as a potential biomarker using blood samples for gastric cancer detection

SUPPLEMENTARY MATERIALS

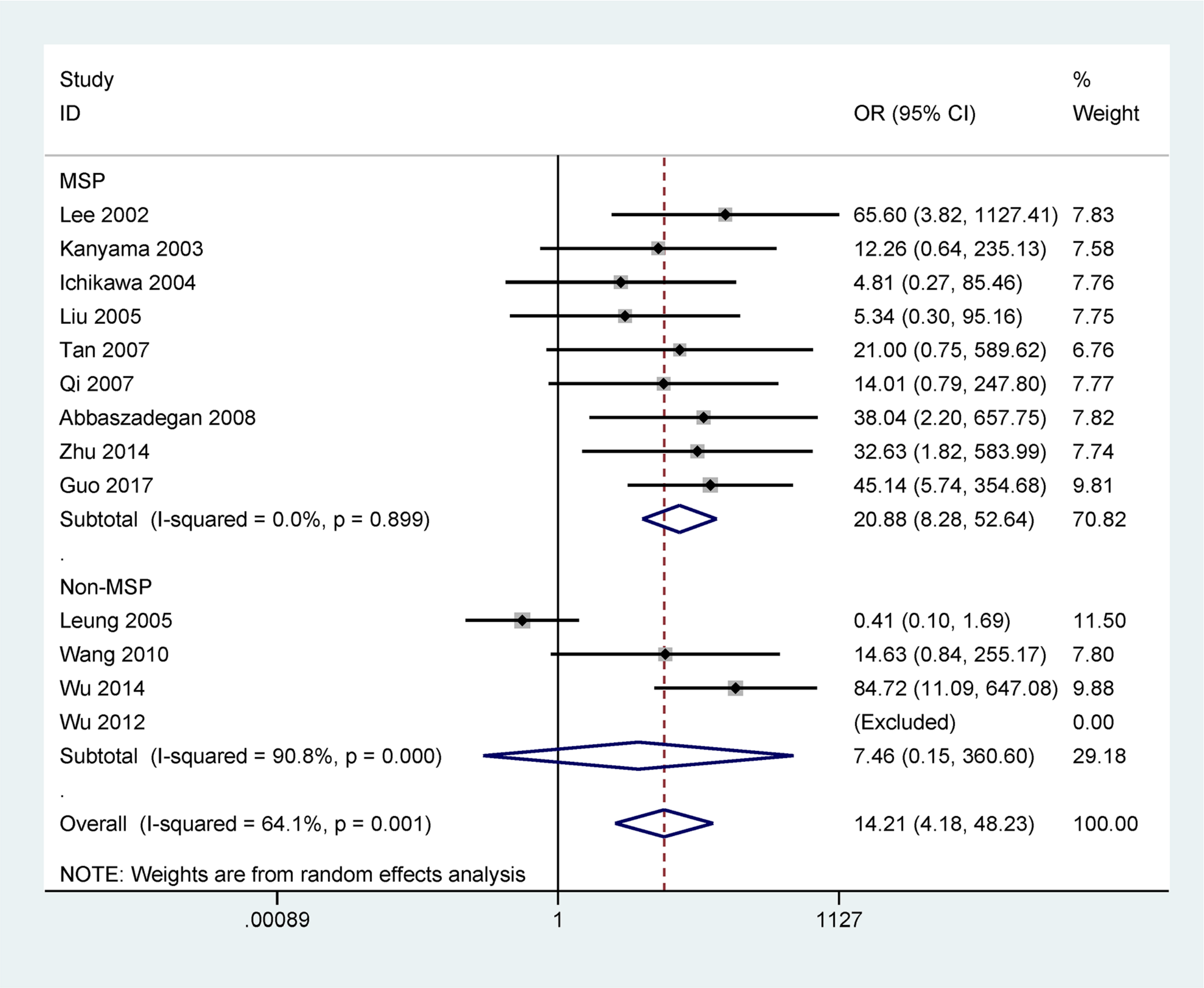

Supplementary Figure 1: Subgroup analysis of *p16* promoter methylation by testing method in the blood of GC vs non-tumor controls.

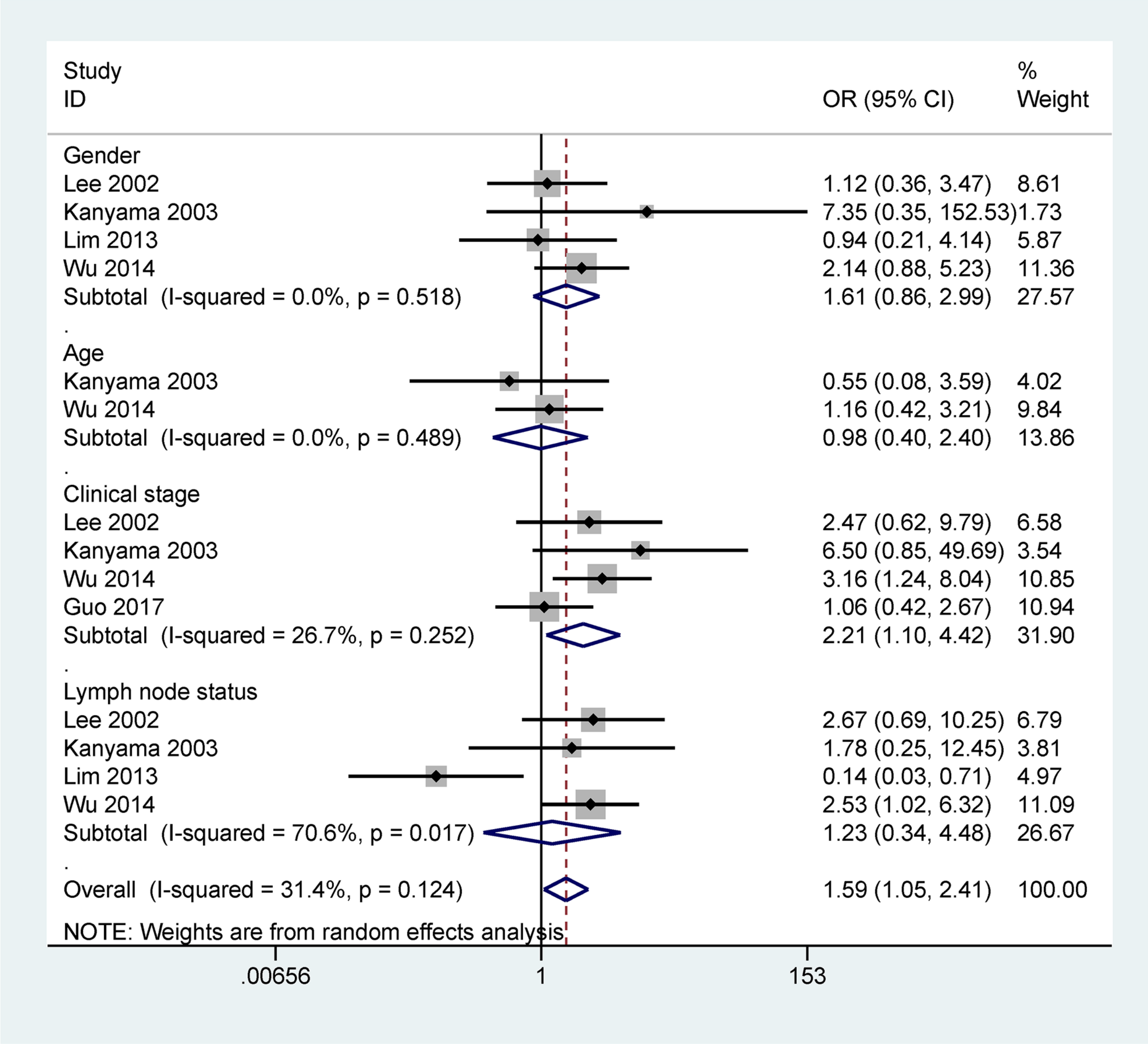

Supplementary Figure 2: Forest plot of the relationship of *p16* promoter methylation with clinicopathological features in blood samples of GC.

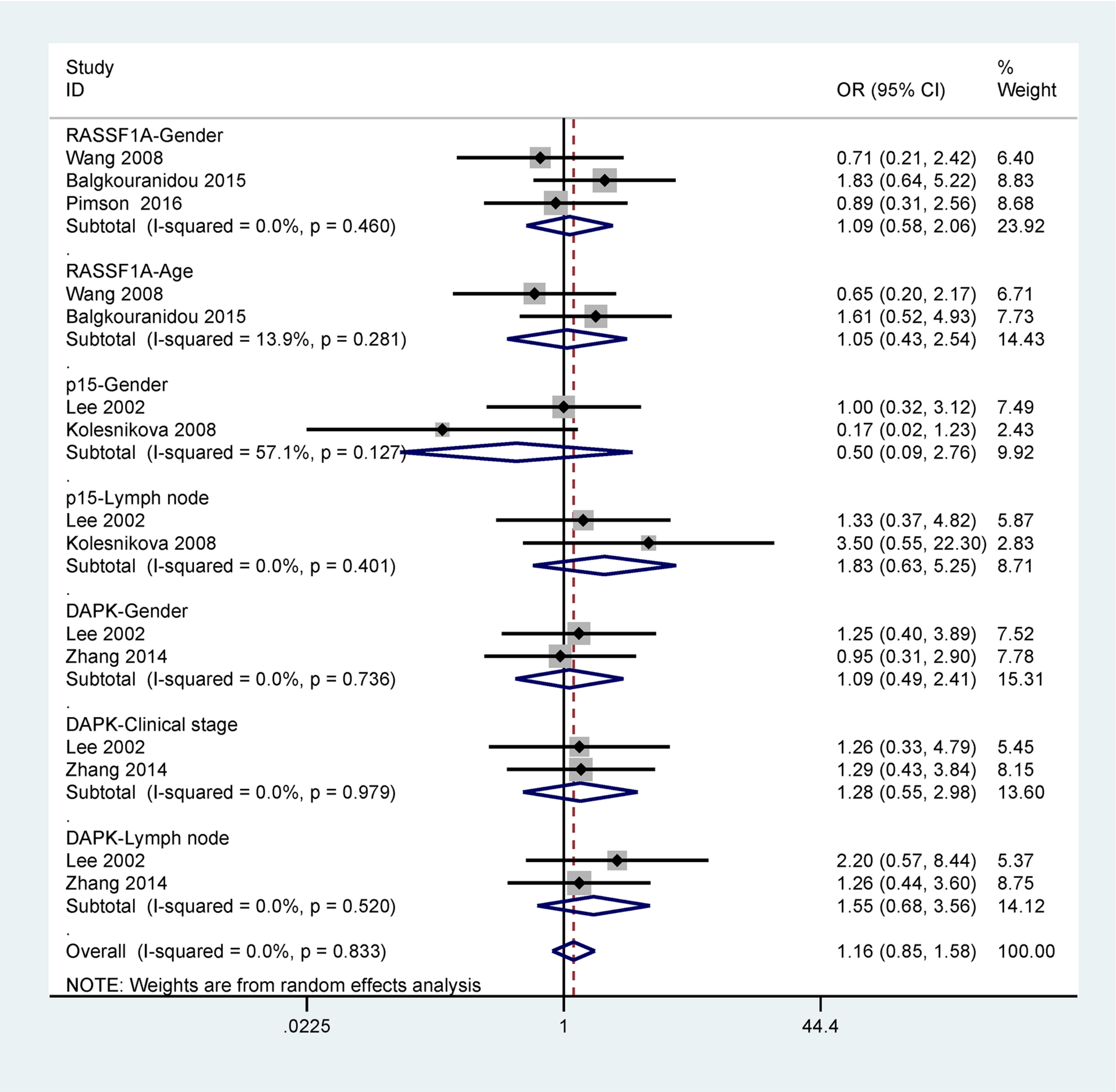

Supplementary Figure 3: Forest plot of the relationship of *RASSF1A, DAPK,* and *p15* promoter methylation with clinicopathological features in blood samples of GC.

Supplementary Table 1 : Baseline characteristics of the included studies of the eligibility

See Supplementary File 1

Supplementary Table 2 : The summary in blood samples of GC patients vs. non-tumor controls

See Supplementary File 2
