# Supplementary material for: Promoter methylation of tumor-related genes as a potential biomarker using blood samples for gastric cancer detection
Source: Oncotarget. 2017 Sep 8;8(44):77783–93. doi: 10.18632/oncotarget.20782 (PMC5652815; doi:10.18632/oncotarget.20782)
Supplement: Supplementary file 2 [file oncotarget-08-77783-s002.docx]

**Supplementary Table 1: Baseline characteristics of the included studies of the eligibility**

| **Gene** | **First author** | **Country** | **Ethnicity** | **Age** | **Method** | **Region** | **Stage** | **GC** | | | **Non-tumor control** | | | **Male** | **Female** | **≥60 years** | **＜60 years** | **Stage 3-4** | **Stage 1-2** | **N+** | **N ^_^** |
| --- | --- | --- | --- | --- | --- | --- | --- | --- | --- | --- | --- | --- | --- | --- | --- | --- | --- | --- | --- | --- | --- |
|  |  |  |  |  |  |  |  | **M%** | **M** | **Total** | **M%** | **M** | **Total** | **M/Total** | **M/Total** | **M/Total** | **M/Total** | **M/Total** | **M/Total** | **M/Total** | **M/Total** |
| *p16* |  |  |  |  |  |  |  |  |  |  |  |  |  |  |  |  |  |  |  |  |  |
|  | Lee 2002 | China | Asians | 62.6 | MSP | Promoter | 1-4 | 51.9 | 28 | 54 | 0 | 0 | 30 | 19/36 | 9/18 |  |  | 24/41 | 4/11 | 24/42 | 4/12 |
|  | Kanyama 2003 | Japan | Asians | NA | MSP | Promoter | 1-4 | 26.1 | 6 | 23 | 0 | 0 | 16 | 6/17 | 0/6 | 3/14 | 3/9 | 4/8 | 2/15 | 4/13 | 2/10 |
|  | Ichikawa 2004 | Japan | Asians | 65 | MSP | Promoter | 1-4 | 18.3 | 20 | 109 | 0 | 0 | 10 |  |  |  |  |  |  |  |  |
|  | Leung 2005 | China | Asians | 66 | QMSP | Promoter | NA | 8.3 | 5 | 60 | 18.2 | 4 | 22 |  |  |  |  |  |  |  |  |
|  | Liu 2005 | China | Asians | NA | MSP | Promoter | 1-4 | 14.3 | 12 | 84 | 0 | 0 | 15 |  |  |  |  |  |  |  |  |
|  | Tan 2007 | Singapore | Asians | NA | MSP | Promoter | NA | 50 | 2 | 4 | 0 | 0 | 10 |  |  |  |  |  |  |  |  |
|  | Qi 2007 | China | Asians | 63.2 | MSP | Promoter | NA | 25 | 13 | 52 | 0 | 0 | 20 |  |  |  |  |  |  |  |  |
|  | Abbaszadegan 2008 | Iran | Caucasians | 64.5 | MSP | Promoter | NA | 26.9 | 14 | 52 | 0 | 0 | 50 |  |  |  |  |  |  |  |  |
|  | Wang 2010 | China | Asians | 51 | NMSP | Promoter | NA | 30.4 | 21 | 69 | 0 | 0 | 16 |  |  |  |  |  |  |  |  |
|  | Wu 2012 | China | Asians | NA | BSP | Promoter | NA | 0 | 0 | 140 | 0 | 0 | 30 |  |  |  |  |  |  |  |  |
|  | Lim 2013 | Korea | Asians | NA | MSP | Promoter | 1-4 | 79.2 | 42 | 53 |  |  |  | 30/38 | 12/15 |  |  |  |  | 16/25 | 26/28 |
|  | Wu 2014 | China | Asians | 53.9 | QMSP | Promoter | 1-4 | 68.5 | 63 | 92 | 2.5 | 1 | 40 | 40/53 | 23/39 | 17/24 | 46/68 | 37/46 | 26/46 | 36/46 | 27/46 |
|  | Zhu 2014 | China | Asians | NA | MSP | Promoter | NA | 34.4 | 11 | 32 | 0 | 0 | 30 |  |  |  |  |  |  |  |  |
|  | Guo 2017 | China | Asians | NA | MSP | Promoter | 1-4 | 72.6 | 77 | 106 | 5.6 | 1 | 18 |  |  |  |  | 54/74 | 23/32 |  |  |
|  |  |  |  |  |  |  |  |  |  |  |  |  |  |  |  |  |  |  |  |  |  |
| *CDH1* |  |  |  |  |  |  |  |  |  |  |  |  |  |  |  |  |  |  |  |  |  |
|  | Lee 2002 | China | Asians | 62.6 | MSP | Promoter | 1-4 | 57.4 | 31 | 54 | 0 | 0 | 30 | 21/36 | 10/18 |  |  | 23/41 | 7/11 | 24/42 | 7/12 |
|  | Ichikawa 2004 | Japan | Asians | 65 | MSP | Promoter | 1-4 | 23.9 | 26 | 109 | 0 | 0 | 10 |  |  |  |  |  |  |  |  |
|  | Leung 2005 | China | Asians | 66 | QMSP | Promoter | NA | 13.3 | 8 | 60 | 0 | 0 | 22 |  |  |  |  |  |  |  |  |
|  | Tan 2007 | Singapore | Asians | NA | MSP | Promoter | NA | 25 | 1 | 4 | 0 | 0 | 10 |  |  |  |  |  |  |  |  |
|  | Qi 2007 | China | Asians | 63.2 | MSP | Promoter | NA | 38.5 | 20 | 52 | 0 | 0 | 20 |  |  |  |  |  |  |  |  |
|  | Bernal 2008 | Chile | Caucasians | NA | MSP | Promoter | NA | 53.5 | 23 | 43 | 6.5 | 2 | 31 |  |  |  |  |  |  |  |  |
|  | Wu 2012 | China | Asians | NA | BSP | Promoter | NA | 0 | 0 | 140 | 0 | 0 | 30 |  |  |  |  |  |  |  |  |
|  | Liu 2012 | China | Asians | 52 | MSP | Promoter | NA | 26.7 | 20 | 75 | 0 | 0 | 40 |  |  |  |  |  |  |  |  |
|  | Zhu 2014 | China | Asians | NA | MSP | Promoter | NA | 28.1 | 9 | 32 | 0 | 0 | 30 |  |  |  |  |  |  |  |  |
|  |  |  |  |  |  |  |  |  |  |  |  |  |  |  |  |  |  |  |  |  |  |
| *RUNX3* |  |  |  |  |  |  |  |  |  |  |  |  |  |  |  |  |  |  |  |  |  |
|  | Tan 2007 | Singapore | Asians | NA | MSP | Promoter | NA | 100 | 4 | 4 | 0 | 0 | 10 |  |  |  |  |  |  |  |  |
|  | Sakakura 2009 | Japan | Asians | NA | QMSP | Promoter | 1-4 | 95.4 | 62 | 65 | 38 | 19 | 50 |  |  |  |  |  |  |  |  |
|  | Zheng 2011 | China | Asians | NA | MSP | Promoter | NA | 45.2 | 28 | 62 | 5 | 2 | 40 | 18/37 | 10/25 | 16/38 | 12/24 | 12/21 | 16/41 | 16/29 | 12/33 |
|  | Lu 2012 | China | Asians | NA | QMSP | Promoter | NA | 70.8 | 143 | 202 | 0.26 | 2 | 772 |  |  |  |  |  |  |  |  |
|  | Liu 2012 | China | Asians | 52 | MSP | Promoter | NA | 37.3 | 28 | 75 | 0 | 0 | 40 |  |  |  |  |  |  |  |  |
|  | Zhu 2014 | China | Asians | NA | MSP | Promoter | NA | 40.6 | 13 | 32 | 3.3 | 1 | 30 |  |  |  |  |  |  |  |  |
|  |  |  |  |  |  |  |  |  |  |  |  |  |  |  |  |  |  |  |  |  |  |
| *MLH1* |  |  |  |  |  |  |  |  |  |  |  |  |  |  |  |  |  |  |  |  |  |
|  | Leung 2005 | China | Asians | 66 | QMSP | Promoter | NA | 41.7 | 25 | 60 | 9.1 | 2 | 22 |  |  |  |  |  |  |  |  |
|  | Qi 2007 | China | Asians | 63.2 | MSP | Promoter | NA | 13.5 | 7 | 52 | 0 | 0 | 20 |  |  |  |  |  |  |  |  |
|  | Kolesnikova 2008 | Russia | Caucasians | NA | MSP | Promoter | NA | 25 | 5 | 20 | 9.1 | 2 | 22 | 4/12 | 1/8 | 1/6 | 4/14 |  |  | 3/11 | 2/9 |
|  | Wu 2012 | China | Asians | NA | BSP | Promoter | NA | 0.7 | 1 | 140 | 0 | 0 | 30 |  |  |  |  |  |  |  |  |
|  | Zhu 2014 | China | Asians | NA | MSP | Promoter | NA | 21.9 | 7 | 32 | 0 | 0 | 30 |  |  |  |  |  |  |  |  |
|  | Liu 2015 | China | Asians | NA | MSP | Promoter | NA | 48 | 24 | 50 | 3.3 | 1 | 30 |  |  |  |  |  |  |  |  |
|  |  |  |  |  |  |  |  |  |  |  |  |  |  |  |  |  |  |  |  |  |  |
| *RASSF1A* |  |  |  |  |  |  |  |  |  |  |  |  |  |  |  |  |  |  |  |  |  |
|  | Tan 2007 | Singapore | Asians | NA | MSP | Promoter | NA | 25 | 1 | 4 | 0 | 0 | 10 |  |  |  |  |  |  |  |  |
|  | Wang 2008 | China | Asians | NA | MSP | Promoter | 1-4 | 34 | 16 | 47 | 1.7 | 1 | 60 | 9/29 | 7/18 | 8/28 | 8/21 |  |  |  |  |
|  | Zhu 2014 | China | Asians | NA | MSP | Promoter | NA | 21.9 | 7 | 32 | 0 | 0 | 30 |  |  |  |  |  |  |  |  |
|  | Balgkouranidou 2015 | Greece | Caucasians | 70.5 | MSP | Promoter | 1-3 | 68.5 | 50 | 73 | 0 | 0 | 20 | 37/51 | 13/22 | 39/56 | 10/17 | 31/38 | 11/20 | 34/43 | 6/15 |
|  | Pimson 2016 | Thailand | Asians | NA | MSP | Promoter | 1-4 | 83.2 | 84 | 101 | 5.4 | 11 | 202 | 47/57 | 37/44 |  |  |  |  |  |  |
|  |  |  |  |  |  |  |  |  |  |  |  |  |  |  |  |  |  |  |  |  |  |
| *p15* |  |  |  |  |  |  |  |  |  |  |  |  |  |  |  |  |  |  |  |  |  |
|  | Lee 2002 | China | Asians | 62.6 | MSP | Promoter | 1-4 | 55.6 | 30 | 54 | 0 | 0 | 30 | 20/36 | 10/18 |  |  | 22/41 | 8/11 | 24/42 | 6/12 |
|  | Leung 2005 | China | Asians | 66 | QMSP | Promoter | NA | 40 | 24 | 60 | 13.6 | 3 | 22 |  |  |  |  |  |  |  |  |
|  | Qi 2007 | China | Asians | 63.2 | MSP | Promoter | NA | 30.8 | 16 | 52 | 0 | 0 | 20 |  |  |  |  |  |  |  |  |
|  | Kolesnikova 2008 | Russia | Caucasians | NA | MSP | Promoter | NA | 50 | 10 | 20 | 18.2 | 4 | 22 | 4/12 | 6/8 | 4/6 | 6/14 |  |  | 7/11 | 3/9 |
|  |  |  |  |  |  |  |  |  |  |  |  |  |  |  |  |  |  |  |  |  |  |
| *APC* |  |  |  |  |  |  |  |  |  |  |  |  |  |  |  |  |  |  |  |  |  |
|  | Leung 2005 | China | Asians | 66 | QMSP | Promoter | NA | 16.7 | 10 | 60 | 0 | 0 | 22 |  |  |  |  |  |  |  |  |
|  | Bernal 2008 | Chile | Caucasians | NA | MSP | Promoter | NA | 76.7 | 33 | 43 | 64.5 | 20 | 31 |  |  |  |  |  |  |  |  |
|  | Liu 2012 | China | Asians | 52 | MSP | Promoter | NA | 30.7 | 23 | 75 | 0 | 0 | 40 |  |  |  |  |  |  |  |  |
|  | Balgkouranidou 2015 | Greece | Caucasians | 70.5 | MSP | Promoter | 1-3 | 83.6 | 61 | 73 | 0 | 0 | 20 | 42/51 | 19/22 | 46/56 | 14/17 | 32/38 | 16/20 | 37/43 | 12/15 |
|  |  |  |  |  |  |  |  |  |  |  |  |  |  |  |  |  |  |  |  |  |  |
| *DAPK* |  |  |  |  |  |  |  |  |  |  |  |  |  |  |  |  |  |  |  |  |  |
|  | Lee 2002 | China | Asians | 62.6 | MSP | Promoter | 1-4 | 48.1 | 26 | 54 | 0 | 0 | 30 | 18/36 | 8/18 |  |  | 21/41 | 5/11 | 22/42 | 4/12 |
|  | Kong 2009 | China | Asians | 62.38 | MSP | Promoter | 1-4 | 16.7 | 11 | 66 | 0 | 0 | 20 |  |  |  |  |  |  |  |  |
|  | Zhang 2014 | China | Asians | 61.49 | MSP | Promoter | 1-4 | 49.1 | 28 | 57 | 28.6 | 12 | 42 | 19/39 | 9/18 | 16/33 | 12/24 | 19/37 | 9/20 | 17/33 | 11/24 |
|  |  |  |  |  |  |  |  |  |  |  |  |  |  |  |  |  |  |  |  |  |  |
| *GSTP1* |  |  |  |  |  |  |  |  |  |  |  |  |  |  |  |  |  |  |  |  |  |
|  | Lee 2002 | China | Asians | 62.6 | MSP | Promoter | 1-4 | 14.8 | 8 | 54 | 0 | 0 | 30 | 6/36 | 2/18 |  |  | 3/41 | 4/11 | 4/42 | 4/12 |
|  | Leung 2005 | China | Asians | 66 | QMSP | Promoter | NA | 3.3 | 2 | 60 | 0 | 0 | 22 |  |  |  |  |  |  |  |  |
|  | Qi 2007 | China | Asians | 63.2 | MSP | Promoter | NA | 15.4 | 8 | 52 | 0 | 0 | 20 |  |  |  |  |  |  |  |  |
|  |  |  |  |  |  |  |  |  |  |  |  |  |  |  |  |  |  |  |  |  |  |
| *Reprimo* |  |  |  |  |  |  |  |  |  |  |  |  |  |  |  |  |  |  |  |  |  |
|  | Bernal 2008 | Chile | Caucasians | NA | MSP | Promoter | NA | 95.3 | 41 | 43 | 9.7 | 3 | 31 |  |  |  |  |  |  |  |  |
|  | Liu 2015 | China | Asians | NA | MSP | Promoter | NA | 62 | 31 | 50 | 0 | 0 | 30 |  |  |  |  |  |  |  |  |
|  | Wang 2016 | China | Asians | NA | MS-MCA | Promoter | NA | 94.3 | 33 | 35 | 17.5 | 10 | 57 |  |  |  |  |  |  |  |  |
|  |  |  |  |  |  |  |  |  | 105 | 128 |  |  |  |  |  |  |  |  |  |  |  |
| *MGMT* |  |  |  |  |  |  |  |  |  |  |  |  |  |  |  |  |  |  |  |  |  |
|  | Leung 2005 | China | Asians | 66 | QMSP | Promoter | NA | 58.3 | 35 | 60 | 36.4 | 8 | 22 |  |  |  |  |  |  |  |  |
|  | Kolesnikova 2008 | Russia | Caucasians | NA | MSP | Promoter | NA | 70 | 14 | 20 | 36.4 | 8 | 22 | 8/12 | 6/8 | 6/6 | 8/14 |  |  | 9/11 | 5/9 |
|  | Wang 2010 | China | Asians | 51 | NMSP | Promoter | NA | 17.4 | 12 | 69 | 0 | 0 | 16 |  |  |  |  |  |  |  |  |
|  |  |  |  |  |  |  |  |  |  |  |  |  |  |  |  |  |  |  |  |  |  |
| *DLEC1* |  |  |  |  |  |  |  |  |  |  |  |  |  |  |  |  |  |  |  |  |  |
|  | Zhang 2010 | China | Asians | NA | MSP | Promoter | 1-4 | 33.8 | 22 | 65 | 3.3 | 2 | 60 |  |  |  |  |  |  |  |  |
|  | Wang 2015 | China | Asians | 63.3 | QMSP | Promoter | 1-4 | 80.5 | 66 | 82 | 7 | 6 | 86 |  |  | 42/54 | 24/28 | 29/34 | 37/48 | 42/53 | 24/29 |
|  |  |  |  |  |  |  |  |  |  |  |  |  |  |  |  |  |  |  |  |  |  |
| *SOCS1* |  |  |  |  |  |  |  |  |  |  |  |  |  |  |  |  |  |  |  |  |  |
|  | Chan 2004 | China | Asians | NA | QMSP | Promoter | NA | 83.3 | 5 | 6 | 16.7 | 1 | 6 |  |  |  |  |  |  |  |  |
|  | Leung 2005 | China | Asians | 66 | QMSP | Promoter | NA | 23.3 | 14 | 60 | 27.3 | 6 | 22 |  |  |  |  |  |  |  |  |
|  |  |  |  |  |  |  |  |  |  |  |  |  |  |  |  |  |  |  |  |  |  |
| *RNF180* |  |  |  |  |  |  |  |  |  |  |  |  |  |  |  |  |  |  |  |  |  |
|  | Cheung 2012 | China | Asians | NA | QMSP | Promoter | NA | 56.3 | 18 | 32 | 0 | 0 | 64 |  |  |  |  |  |  |  |  |
|  | Zhang 2014 | China | Asians | 61.49 | MSP | Promoter | 1-4 | 57.9 | 33 | 57 | 23.8 | 10 | 42 | 20/39 | 13/18 | 20/33 | 13/24 | 27/37 | 6/20 | 24/33 | 9/24 |
|  |  |  |  |  |  |  |  |  |  |  |  |  |  |  |  |  |  |  |  |  |  |
| *TFPI2* |  |  |  |  |  |  |  |  |  |  |  |  |  |  |  |  |  |  |  |  |  |
|  | Hibi 2011 | Japan | Asians | 51-85 | QMSP | NA | 1-4 | 9.6 | 7 | 73 | 0 | 0 | 20 | 7/57 | 0/16 |  |  | 6/40 | 1/33 | 7/42 | 0/31 |
|  | Zhu 2014 | China | Asians | NA | MSP | Promoter | NA | 28.1 | 9 | 32 | 0 | 0 | 30 |  |  |  |  |  |  |  |  |
|  |  |  |  |  |  |  |  |  |  |  |  |  |  |  |  |  |  |  |  |  |  |
| *SFRP2* |  |  |  |  |  |  |  |  |  |  |  |  |  |  |  |  |  |  |  |  |  |
|  | Cheng 2007 | China | Asians | NA | QMSP | Promoter | NA | 66.7 | 12 | 18 | 0 | 0 | 18 |  |  |  |  |  |  |  |  |
|  | Zhang 2014 | China | Asians | 61.49 | MSP | Promoter | 1-4 | 71.9 | 41 | 57 | 42.9 | 18 | 42 | 27/39 | 14/18 | 25/33 | 16/24 | 26/37 | 15/20 | 23/33 | 18/24 |
|  |  |  |  |  |  |  |  |  |  |  |  |  |  |  |  |  |  |  |  |  |  |
| *SFRP1* |  |  |  |  |  |  |  |  |  |  |  |  |  |  |  |  |  |  |  |  |  |
|  | Liu 2012 | China | Asians | 52 | MSP | Promoter | NA | 33.3 | 25 | 75 | 0 | 0 | 40 |  |  |  |  |  |  |  |  |
|  | Liu 2015 | China | Asians | NA | MSP | Promoter | 1-4 | 31 | 13 | 42 | 6.8 | 3 | 44 | 8/30 | 5/12 | 11/25 | 2/17 | 11/33 | 2/9 |  |  |
|  |  |  |  |  |  |  |  |  |  |  |  |  |  |  |  |  |  |  |  |  |  |
| *TIMP3* |  |  |  |  |  |  |  |  |  |  |  |  |  |  |  |  |  |  |  |  |  |
|  | Leung 2005 | China | Asians | 66 | QMSP | Promoter | NA | 16.7 | 10 | 60 | 0 | 0 | 22 |  |  |  |  |  |  |  |  |
|  | Yu 2014 | China | Asians | NA | QMSP | Promoter | 1-4 | 58.7 | 54 | 92 |  |  |  | 32/53 | 22/39 | 11/24 | 43/68 | 46/46 | 8/46 | 44/46 | 10/46 |
|  |  |  |  |  |  |  |  |  |  |  |  |  |  |  |  |  |  |  |  |  |  |
| *RARb* |  |  |  |  |  |  |  |  |  |  |  |  |  |  |  |  |  |  |  |  |  |
|  | Koike 2005 | Japan | Asians | NA | MSP | Promoter | 1-4 | 17.5 | 11 | 63 | 0 | 0 | 10 |  |  |  |  |  |  |  |  |
|  |  |  |  |  |  |  |  |  |  |  |  |  |  |  |  |  |  |  |  |  |  |
| *TGF-betaRII* | |  |  |  |  |  |  |  |  |  |  |  |  |  |  |  |  |  |  |  |  |
|  | Leung 2005 | China | Asians | 66 | QMSP | Promoter | NA | 0 | 0 | 60 | 0 | 0 | 22 |  |  |  |  |  |  |  |  |
|  |  |  |  |  |  |  |  |  |  |  |  |  |  |  |  |  |  |  |  |  |  |
| *SHP1* |  |  |  |  |  |  |  |  |  |  |  |  |  |  |  |  |  |  |  |  |  |
|  | Bernal 2008 | Chile | Caucasians | NA | MSP | Promoter | NA | 0 | 0 | 43 | 0 | 0 | 31 |  |  |  |  |  |  |  |  |
|  |  |  |  |  |  |  |  |  |  |  |  |  |  |  |  |  |  |  |  |  |  |
| *ER* |  |  |  |  |  |  |  |  |  |  |  |  |  |  |  |  |  |  |  |  |  |
|  | Bernal 2008 | Chile | Caucasians | NA | MSP | Promoter | NA | 25.6 | 11 | 43 | 0 | 0 | 31 |  |  |  |  |  |  |  |  |
|  |  |  |  |  |  |  |  |  |  |  |  |  |  |  |  |  |  |  |  |  |  |
| *SEMA3B* |  |  |  |  |  |  |  |  |  |  |  |  |  |  |  |  |  |  |  |  |  |
|  | Bernal 2008 | Chile | Caucasians | NA | MSP | Promoter | NA | 37.2 | 16 | 43 | 0 | 0 | 31 |  |  |  |  |  |  |  |  |
|  |  |  |  |  |  |  |  |  |  |  |  |  |  |  |  |  |  |  |  |  |  |
| *3OST2* |  |  |  |  |  |  |  |  |  |  |  |  |  |  |  |  |  |  |  |  |  |
|  | Bernal 2008 | Chile | Caucasians | NA | MSP | Promoter | NA | 25.6 | 11 | 43 | 0 | 0 | 31 |  |  |  |  |  |  |  |  |
|  |  |  |  |  |  |  |  |  |  |  |  |  |  |  |  |  |  |  |  |  |  |
| *HSulf-1* |  |  |  |  |  |  |  |  |  |  |  |  |  |  |  |  |  |  |  |  |  |
|  | Chen 2009 | China | Asians | 61 | MSP | Promoter | 1-4 | 55 | 11 | 20 | 19 | 4 | 21 |  |  |  |  |  |  |  |  |
|  |  |  |  |  |  |  |  |  |  |  |  |  |  |  |  |  |  |  |  |  |  |
| *IRX1* |  |  |  |  |  |  |  |  |  |  |  |  |  |  |  |  |  |  |  |  |  |
|  | Guo 2010 | China | Asians | NA | MSP | Promoter | NA | 73.3 | 11 | 15 | 10 | 1 | 10 |  |  |  |  |  |  |  |  |
|  |  |  |  |  |  |  |  |  |  |  |  |  |  |  |  |  |  |  |  |  |  |
| *HLTF* |  |  |  |  |  |  |  |  |  |  |  |  |  |  |  |  |  |  |  |  |  |
|  | Guo 2011 | China | Asians | 57.8 | NMSP | Promoter | 1-4 | 20.8 | 20 | 96 | 0.8 | 1 | 122 |  |  |  |  |  |  |  |  |
|  |  |  |  |  |  |  |  |  |  |  |  |  |  |  |  |  |  |  |  |  |  |
| *SLC19A3* |  |  |  |  |  |  |  |  |  |  |  |  |  |  |  |  |  |  |  |  |  |
|  | Ng 2011 | China | Asians | NA | QMSP | Promoter | NA | 85 | 17 | 20 | 15 | 3 | 20 |  |  |  |  |  |  |  |  |
|  |  |  |  |  |  |  |  |  |  |  |  |  |  |  |  |  |  |  |  |  |  |
| *CHRM2* |  |  |  |  |  |  |  |  |  |  |  |  |  |  |  |  |  |  |  |  |  |
|  | Chen 2012 | China | Asians | 62 | MSP | Promoter | 1-4 | 31 | 18 | 58 | 11.8 | 9 | 76 |  |  |  |  |  |  |  |  |
|  |  |  |  |  |  |  |  |  |  |  |  |  |  |  |  |  |  |  |  |  |  |
| *FAM5C* |  |  |  |  |  |  |  |  |  |  |  |  |  |  |  |  |  |  |  |  |  |
|  | Chen 2012 | China | Asians | 62 | MSP | Promoter | 1-4 | 31 | 18 | 58 | 5.3 | 4 | 76 |  |  |  |  |  |  |  |  |
|  |  |  |  |  |  |  |  |  |  |  |  |  |  |  |  |  |  |  |  |  |  |
| *MYLK* |  |  |  |  |  |  |  |  |  |  |  |  |  |  |  |  |  |  |  |  |  |
|  | Chen 2012 | China | Asians | 62 | MSP | Promoter | 1-4 | 70.7 | 41 | 58 | 19.7 | 15 | 76 |  |  |  |  |  |  |  |  |
|  |  |  |  |  |  |  |  |  |  |  |  |  |  |  |  |  |  |  |  |  |  |
| *WIF-1* |  |  |  |  |  |  |  |  |  |  |  |  |  |  |  |  |  |  |  |  |  |
|  | Liu 2012 | China | Asians | 52 | MSP | Promoter | NA | 34.7 | 26 | 75 | 0 | 0 | 40 |  |  |  |  |  |  |  |  |
|  |  |  |  |  |  |  |  |  |  |  |  |  |  |  |  |  |  |  |  |  |  |
| *DLC-1* |  |  |  |  |  |  |  |  |  |  |  |  |  |  |  |  |  |  |  |  |  |
|  | Liu 2012 | China | Asians | 52 | MSP | Promoter | NA | 29.3 | 22 | 75 | 0 | 0 | 40 |  |  |  |  |  |  |  |  |
|  |  |  |  |  |  |  |  |  |  |  |  |  |  |  |  |  |  |  |  |  |  |
| *DKK* |  |  |  |  |  |  |  |  |  |  |  |  |  |  |  |  |  |  |  |  |  |
|  | Liu 2012 | China | Asians | 52 | MSP | Promoter | NA | 32 | 24 | 75 | 0 | 0 | 40 |  |  |  |  |  |  |  |  |
|  |  |  |  |  |  |  |  |  |  |  |  |  |  |  |  |  |  |  |  |  |  |
| *SOX17* |  |  |  |  |  |  |  |  |  |  |  |  |  |  |  |  |  |  |  |  |  |
|  | Balgkouranidou 2013 | Greece | Caucasians | 67.07 | MSP | Promoter | NA | 58.9 | 43 | 73 | 0 | 0 | 20 | 28/51 | 15/22 | 30/56 | 12/16 |  |  | 28/43 | 9/15 |
|  |  |  |  |  |  |  |  |  |  |  |  |  |  |  |  |  |  |  |  |  |  |
| *SEPT9* |  |  |  |  |  |  |  |  |  |  |  |  |  |  |  |  |  |  |  |  |  |
|  | Lee 2013 | Korea | Asians | NA | RTPCR | NA | 1-4 | 17.6 | 27 | 153 | 9.4 | 9 | 96 | 19/91 | 8/62 |  |  | 14/58 | 13/95 |  |  |
|  |  |  |  |  |  |  |  |  |  |  |  |  |  |  |  |  |  |  |  |  |  |
| *XAF1* |  |  |  |  |  |  |  |  |  |  |  |  |  |  |  |  |  |  |  |  |  |
|  | Ling 2013 | China | Asians | NA | QMSP | Promoter | 1-4 | 69.8 | 141 | 202 | 0 | 0 | 88 | 83/120 | 58/82 | 36/57 | 105/145 | 130/138 | 11/64 | 124/134 | 17/68 |
|  |  |  |  |  |  |  |  |  |  |  |  |  |  |  |  |  |  |  |  |  |  |
| *BCL6B* |  |  |  |  |  |  |  |  |  |  |  |  |  |  |  |  |  |  |  |  |  |
|  | Yang 2013 | China | Asians | NA | BGS | Promoter | 4 | 42.5 | 17 | 40 | 0 | 0 | 22 | 13/33 | 4/7 |  |  |  |  |  |  |
|  |  |  |  |  |  |  |  |  |  |  |  |  |  |  |  |  |  |  |  |  |  |
| *MINT2* |  |  |  |  |  |  |  |  |  |  |  |  |  |  |  |  |  |  |  |  |  |
|  | Han 2014 | China | Asians | NA | QMSP | Promoter | 1-4 | 39.1 | 36 | 92 | 3.4 | 3 | 88 | 24/53 | 12/39 | 8/24 | 28/68 | 29/46 | 7/46 | 29/46 | 7/46 |
|  |  |  |  |  |  |  |  |  |  |  |  |  |  |  |  |  |  |  |  |  |  |
| *Zic1* |  |  |  |  |  |  |  |  |  |  |  |  |  |  |  |  |  |  |  |  |  |
|  | Chen 2015 | China | Asians | 59.8 | MSP | Promoter | 0-4 | 60.6 | 63 | 104 | 0 | 0 | 20 |  |  |  |  | 27/45 | 19/31 | 31/48 | 19/35 |
|  |  |  |  |  |  |  |  |  |  |  |  |  |  |  |  |  |  |  |  |  |  |
| *FLNC* |  |  |  |  |  |  |  |  |  |  |  |  |  |  |  |  |  |  |  |  |  |
|  | Wang 2015 | China | Asians | 63.3 | QMSP | Promoter | 1-4 | 67.1 | 55 | 82 | 7 | 6 | 86 |  |  | 37/54 | 18/28 | 26/34 | 29/48 | 38/53 | 17/29 |
|  |  |  |  |  |  |  |  |  |  |  |  |  |  |  |  |  |  |  |  |  |  |
| *THBS1* |  |  |  |  |  |  |  |  |  |  |  |  |  |  |  |  |  |  |  |  |  |
|  | Wang 2015 | China | Asians | 63.3 | QMSP | Promoter | 1-4 | 63.4 | 52 | 82 | 5.8 | 5 | 86 |  |  | 39/54 | 13/28 | 24/34 | 28/48 | 34/53 | 18/29 |
|  |  |  |  |  |  |  |  |  |  |  |  |  |  |  |  |  |  |  |  |  |  |
| *UCHL1* |  |  |  |  |  |  |  |  |  |  |  |  |  |  |  |  |  |  |  |  |  |
|  | Wang 2015 | China | Asians | 63.3 | QMSP | Promoter | 1-4 | 56.1 | 46 | 82 | 10.5 | 9 | 86 |  |  | 32/54 | 14/28 | 24/34 | 22/48 | 35/53 | 11/29 |
|  |  |  |  |  |  |  |  |  |  |  |  |  |  |  |  |  |  |  |  |  |  |
| *RASSF10* |  |  |  |  |  |  |  |  |  |  |  |  |  |  |  |  |  |  |  |  |  |
|  | Xue 2016 | China | Asians | NA | BSP | Promoter | 1-4 | 81.7 | 67 | 82 | 10 | 5 | 50 |  |  |  |  |  |  |  |  |
|  |  |  |  |  |  |  |  |  |  |  |  |  |  |  |  |  |  |  |  |  |  |
| *OSR2* |  |  |  |  |  |  |  |  |  |  |  |  |  |  |  |  |  |  |  |  |  |
|  | Li 2016 | China | Asians | 56.75 | MSP | Promoter | 1-4 | 62.5 | 30 | 48 | 8 | 2 | 25 | 23/39 | 7/9 | 14/22 | 16/26 | 17/26 | 13/22 | 21/32 | 9/16 |
|  |  |  |  |  |  |  |  |  |  |  |  |  |  |  |  |  |  |  |  |  |  |
| *VAV3* |  |  |  |  |  |  |  |  |  |  |  |  |  |  |  |  |  |  |  |  |  |
|  | Li 2016 | China | Asians | 56.75 | MSP | Promoter | 1-4 | 45.8 | 22 | 48 | 0 | 0 | 25 | 19/39 | 3/9 | 9/22 | 13/26 | 14/26 | 8/22 | 17/32 | 5/16 |
|  |  |  |  |  |  |  |  |  |  |  |  |  |  |  |  |  |  |  |  |  |  |
| *PPFIA3* |  |  |  |  |  |  |  |  |  |  |  |  |  |  |  |  |  |  |  |  |  |
|  | Li 2016 | China | Asians | 56.75 | MSP | Promoter | 1-4 | 56.3 | 27 | 48 | 4 | 1 | 25 | 23/39 | 4/9 | 13/22 | 14/26 | 17/26 | 10/22 | 19/32 | 8/16 |
|  |  |  |  |  |  |  |  |  |  |  |  |  |  |  |  |  |  |  |  |  |  |
| *PCDH10* |  |  |  |  |  |  |  |  |  |  |  |  |  |  |  |  |  |  |  |  |  |
|  | Pimson 2016 | Thailand | Asians | NA | MSP | Promoter | 1-4 | 94.1 | 95 | 101 | 3 | 6 | 202 | 53/57 | 42/44 |  |  |  |  |  |  |
|  |  |  |  |  |  |  |  |  |  |  |  |  |  |  |  |  |  |  |  |  |  |
| *Survivin* |  |  |  |  |  |  |  |  |  |  |  |  |  |  |  |  |  |  |  |  |  |
|  | Guo 2017 | China | Asians | NA | MSP | Promoter | 1-4 | 6.6 | 7 | 106 | 0 | 0 | 18 |  |  |  |  | 4/74 | 3/32 |  |  |
|  |  |  |  |  |  |  |  |  |  |  |  |  |  |  |  |  |  |  |  |  |  |
| *Rb* |  |  |  |  |  |  |  |  |  |  |  |  |  |  |  |  |  |  |  |  |  |
|  | Guo 2017 | China | Asians | NA | MSP | Promoter | 1-4 | 17.9 | 19 | 106 | 5.6 | 1 | 18 |  |  |  |  | 15/74 | 4/32 |  |  |
|  |  |  |  |  |  |  |  |  |  |  |  |  |  |  |  |  |  |  |  |  |  |
| *SPG20* |  |  |  |  |  |  |  |  |  |  |  |  |  |  |  |  |  |  |  |  |  |
|  | Zhang 2014 | China | Asians | 61.2 | MSP | Promoter | 1-3 | 48.8 | 20 | 41 | 0 | 0 | 21 | 14/30 | 6/11 |  |  | 11/25 | 9/16 | 16/31 | 4/10 |

NA: not applicable; MSP: methylation-specific polymerase chain reaction; QMSP: quantitative methylation-specific polymerase chain reaction; BSP: bisulfite DNA sequencing; NMSP: nested methylation-specific polymerase chain reaction; MS-MCA: methylation-sensitive melt curve analysis; RTPCR: real-time polymerase chain reaction; BGS: bisulfite genomic sequence; M: methylation; N: lymph node status; Total: sample size.
