# Supplementary material for: Promoter methylation of tumor-related genes as a potential biomarker using blood samples for gastric cancer detection
Source: Oncotarget. 2017 Sep 8;8(44):77783–93. doi: 10.18632/oncotarget.20782 (PMC5652815; doi:10.18632/oncotarget.20782)
Supplement: Supplementary file 3 [file oncotarget-08-77783-s003.docx]

**Supplementary** **Table 2: The summary in blood samples of GC patients vs. non-tumor controls**

| ***Gene*** | **Studies** | **Overall OR (95 CI%)** | ***I^2^*; *p*** | ***P* value** | **Cases** | **Controls** | **Case (M %)** | **Control (M %)** |
| --- | --- | --- | --- | --- | --- | --- | --- | --- |
| *p16* | 13 | 14.21 (4.18-48.23) | 64.1%; 0.001 | < 0.001 | 877 | 307 | 31.0 | 2.0 |
| *CDH1* | 9 | 18.19 (7.38-44.80) | 0.0%; 0.935 | < 0.001 | 569 | 223 | 24.3 | 0.9 |
| *RUNX3* | 6 | 63.66 (13.42-302.02) | 75.0%; 0.001 | < 0.001 | 440 | 942 | 63.2 | 2.5 |
| *MLH1* | 6 | 6.81 (2.84-16.35) | 0.0%; 0.423 | < 0.001 | 354 | 154 | 19.5 | 3.2 |
| *RASSF1A* | 5 | 64.15 (32.29-127.47) | 0.0%; 0.533 | < 0.001 | 257 | 322 | 61.5 | 3.7 |
| *p15* | 4 | 7.92 (2.41-26.09) | 37.6%; 0.187 | 0.001 | 186 | 94 | 43.0 | 7.4 |
| *APC* | 4 | 15.60 (1.24-196.14) | 79.7%; 0.002 | 0.033 | 251 | 113 | 50.6 | 17.7 |
| *DAPK* | 3 | 7.82 (0.92-66.26) | 66.2%; 0.052 | 0.059 | 177 | 92 | ND | ND |
| *GSTP1* | 3 | 5.75 (1.05-31.62) | 0.0%; 0.685 | 0.044 | 166 | 72 | 10.8 | 0.0 |
| *Reprimo* | 3 | 111.10 (36.67-336.59) | 0.0%; 0.762 | < 0.001 | 128 | 118 | 82.0 | 11.0 |
| *MGMT* | 3 | 3.16 (1.47-6.81) | 0.0%; 0.697 | 0.003 | 149 | 60 | 40.9 | 26.7 |
| *DLEC1* | 2 | 32.26 (8.95-116.30) | 52.5%; 0.147 | < 0.001 | 147 | 146 | ND | ND |
| *SOCS1* | 2 | 3.34 (0.12-93.62) | 77.2%; 0.036 | 0.478 | 66 | 28 | ND | ND |
| *RNF180* | 2 | 21.41 (0.48-960.82) | 84.7%; 0.011 | 0.114 | 89 | 106 | ND | ND |
| *TFPI2* | 2 | 10.71 (1.38-83.25) | 0%; 0.422 | 0.023 | 105 | 50 | ND | ND |
| *SFRP2* | 2 | 11.31 (0.55-231.48) | 75.1%; 0.045 | 0.115 | 75 | 60 | ND | ND |
| *SFRP1* | 2 | 11.16 (1.65-75.35) | 41.8%; 0.190 | 0.013 | 117 | 84 | ND | ND |
| *TIMP3* | 1 | 9.36 (0.53-166.71) | NA | 0.128 | 60 | 22 | ND | ND |
| *RARb* | 1 | 4.60 (0.25-84.27) | NA | 0.304 | 63 | 10 | ND | ND |
| *TGF-betaRII* | 1 | NA | NA | NA | 60 | 22 | ND | ND |
| *SHP1* | 1 | NA | NA | NA | 43 | 31 | ND | ND |
| *ER* | 1 | 22.29 (1.26-394.56) | NA | 0.034 | 43 | 31 | ND | ND |
| *SEMA3B* | 1 | 37.80 (2.17-659.79) | NA | 0.013 | 43 | 31 | ND | ND |
| *3OST2* | 1 | 22.29 (1.26-394.56) | NA | 0.034 | 43 | 31 | ND | ND |
| *HSulf-1* | 1 | 5.19 (1.28-21.08) | NA | 0.021 | 20 | 21 | ND | ND |
| *IRX1* | 1 | 24.75 (2.33-262.59) | NA | 0.008 | 15 | 10 | ND | ND |
| *HLTF* | 1 | 31.84 (4.19-242.15) | NA | 0.001 | 96 | 122 | ND | ND |
| *SLC19A3* | 1 | 32.11 (5.66-182.18) | NA | < 0.001 | 20 | 20 | ND | ND |
| *CHRM2* | 1 | 3.35 (1.37-8.16) | NA | 0.008 | 58 | 76 | ND | ND |
| *FAM5C* | 1 | 8.10 (2.56-25.59) | NA | < 0.001 | 58 | 76 | ND | ND |
| *MYLK* | 1 | 9.81 (4.41-21.81) | NA | < 0.001 | 58 | 76 | ND | ND |
| *WIF-1* | 1 | 43.36 (2.56-733.71) | NA | 0.009 | 75 | 40 | ND | ND |
| *DLC-1* | 1 | 34.07 (2.01-578.42) | NA | 0.015 | 75 | 40 | ND | ND |
| *DKK* | 1 | 38.53 (2.27-653.01) | NA | 0.011 | 75 | 40 | ND | ND |
| *SOX17* | 1 | 58.48 (3.41-1004.20) | NA | 0.005 | 73 | 20 | ND | ND |
| *SEPT9* | 1 | 2.07 (0.93-4.62) | NA | 0.075 | 153 | 96 | ND | ND |
| *XAF1* | 1 | 407.24 (24.87-6668.24) | NA | < 0.001 | 202 | 88 | ND | ND |
| *BCL6B* | 1 | 33.51 (1.90-591.00) | NA | 0.016 | 40 | 22 | ND | ND |
| *MINT2* | 1 | 18.21 (5.35-62.01) | NA | < 0.001 | 92 | 88 | ND | ND |
| *Zic1* | 1 | 62.73 (3.69-1065.79) | NA | 0.004 | 104 | 20 | ND | ND |
| *FLNC* | 1 | 27.16 (10.52-70.15) | NA | < 0.001 | 82 | 86 | ND | ND |
| *THBS1* | 1 | 28.08 (10.24-77.00) | NA | < 0.001 | 82 | 86 | ND | ND |
| *UCHL1* | 1 | 10.93 (4.83-24.74) | NA | < 0.001 | 82 | 86 | ND | ND |
| *RASSF10* | 1 | 40.20 (13.65-118.41) | NA | < 0.001 | 82 | 50 | ND | ND |
| *OSR2* | 1 | 19.17 (4.03-91.08) | NA | < 0.001 | 48 | 25 | ND | ND |
| *VAV3* | 1 | 43.30 (2.49-752.1) | NA | 0.01 | 48 | 25 | ND | ND |
| *PPFIA3* | 1 | 30.86 (3.85-247.01) | NA | 0.001 | 48 | 25 | ND | ND |
| *PCDH10* | 1 | 517.22 (162.50-1646.29) | NA | < 0.001 | 101 | 202 | ND | ND |
| *Survivin* | 1 | 2.79 (0.15-50.96) | NA | 0.489 | 106 | 18 | ND | ND |
| *Rb* | 1 | 3.71 (0.47-29.63) | NA | 0.216 | 106 | 18 | ND | ND |
| *SPG20* | 1 | 41.00 (2.33-721.95) | NA | 0.011 | 41 | 21 | ND | ND |

GC: gastric cancer; OR: odds ratio; 95% CI: 95% confidence interval; M: methylation; NA: not applicable; ND: not done.
